# Synthesizing images of tau pathology from cross-modal neuroimaging using deep learning

**DOI:** 10.1093/brain/awad346

**Published:** 2023-10-07

**Authors:** Jeyeon Lee, Brian J Burkett, Hoon-Ki Min, Matthew L Senjem, Ellen Dicks, Nick Corriveau-Lecavalier, Carly T Mester, Heather J Wiste, Emily S Lundt, Melissa E Murray, Aivi T Nguyen, Ross R Reichard, Hugo Botha, Jonathan Graff-Radford, Leland R Barnard, Jeffrey L Gunter, Christopher G Schwarz, Kejal Kantarci, David S Knopman, Bradley F Boeve, Val J Lowe, Ronald C Petersen, Clifford R Jack, David T Jones

**Affiliations:** Department of Radiology, Mayo Clinic, Rochester, MN 55905, USA; Department of Biomedical Engineering, Hanyang University, Seoul 04763, Korea; Department of Radiology, Mayo Clinic, Rochester, MN 55905, USA; Department of Radiology, Mayo Clinic, Rochester, MN 55905, USA; Department of Information Technology, Mayo Clinic, Rochester, MN 55905, USA; Department of Neurology, Mayo Clinic, Rochester, MN 55905, USA; Department of Neurology, Mayo Clinic, Rochester, MN 55905, USA; Department of Health Sciences Research, Mayo Clinic, Rochester, MN 55905, USA; Department of Health Sciences Research, Mayo Clinic, Rochester, MN 55905, USA; Department of Health Sciences Research, Mayo Clinic, Rochester, MN 55905, USA; Department of Neuroscience, Mayo Clinic, Jacksonville, FL 32224, USA; Department of Laboratory Medicine and Pathology, Mayo Clinic, Rochester, MN 55905, USA; Department of Laboratory Medicine and Pathology, Mayo Clinic, Rochester, MN 55905, USA; Department of Neurology, Mayo Clinic, Rochester, MN 55905, USA; Department of Neurology, Mayo Clinic, Rochester, MN 55905, USA; Department of Neurology, Mayo Clinic, Rochester, MN 55905, USA; Department of Radiology, Mayo Clinic, Rochester, MN 55905, USA; Department of Radiology, Mayo Clinic, Rochester, MN 55905, USA; Department of Radiology, Mayo Clinic, Rochester, MN 55905, USA; Department of Neurology, Mayo Clinic, Rochester, MN 55905, USA; Department of Neurology, Mayo Clinic, Rochester, MN 55905, USA; Department of Radiology, Mayo Clinic, Rochester, MN 55905, USA; Department of Neurology, Mayo Clinic, Rochester, MN 55905, USA; Department of Radiology, Mayo Clinic, Rochester, MN 55905, USA; Department of Radiology, Mayo Clinic, Rochester, MN 55905, USA; Department of Neurology, Mayo Clinic, Rochester, MN 55905, USA

**Keywords:** tau PET, FDG PET, Alzheimer’s disease, deep learning, cross-modality imputation

## Abstract

Given the prevalence of dementia and the development of pathology-specific disease-modifying therapies, high-value biomarker strategies to inform medical decision-making are critical. *In vivo* tau-PET is an ideal target as a biomarker for Alzheimer’s disease diagnosis and treatment outcome measure. However, tau-PET is not currently widely accessible to patients compared to other neuroimaging methods. In this study, we present a convolutional neural network (CNN) model that imputes tau-PET images from more widely available cross-modality imaging inputs. Participants (*n* = 1192) with brain T_1_-weighted MRI (T1w), fluorodeoxyglucose (FDG)-PET, amyloid-PET and tau-PET were included. We found that a CNN model can impute tau-PET images with high accuracy, the highest being for the FDG-based model followed by amyloid-PET and T1w. In testing implications of artificial intelligence-imputed tau-PET, only the FDG-based model showed a significant improvement of performance in classifying tau positivity and diagnostic groups compared to the original input data, suggesting that application of the model could enhance the utility of the metabolic images. The interpretability experiment revealed that the FDG- and T1w-based models utilized the non-local input from physically remote regions of interest to estimate the tau-PET, but this was not the case for the Pittsburgh compound B-based model. This implies that the model can learn the distinct biological relationship between FDG-PET, T1w and tau-PET from the relationship between amyloid-PET and tau-PET. Our study suggests that extending neuroimaging’s use with artificial intelligence to predict protein specific pathologies has great potential to inform emerging care models.

## Teaser

Tau PET is an attractive biomarker for Alzheimer’s disease but is not readily accessible to patients. Lee *et al.* present an artificial intelligence model that can impute tau PET images from more widely available cross-modality imaging inputs, and propose that the model could help support clinical decision-making.

## Introduction

Together with amyloid-β (Aβ) plaques, misfolded tau neurofibrillary tangles (NFT) are the characteristic pathologic feature of tauopathies, a group of progressive neurodegenerative disease entities including Alzheimer’s disease.^1,2^ Tau-PET, which is a minimal invasive method to quantify the extent and distribution of NFT in the brain,^3-5^ is therefore a promising tool to assess response to therapy or changes over time.^6^ Cross-sectional studies showed that tau-PET uptake levels can be used effectively to support a clinical diagnosis of Alzheimer’s disease dementia and to estimate disease severity.^7-12^ Tau-PET uptake patterns have been associated with specific clinical phenotypes of Alzheimer’s disease, whereas amyloid-PET has not, with distinct distributions of tau pathology associated with posterior cortical atrophy, logopenic variant primary progressive aphasia, and other presentations of Alzheimer’s disease.^8,13-17^

Accordingly, multiple tau-PET agents have been developed for both Alzheimer’s disease and other taupathies.^4^ Recently, ^18^F-flortaucipir (^18^F-AV-1451) received FDA approval for clinical use in the evaluation of Alzheimer’s disease.^18^ This ligand has been shown to have specificity for Alzheimer’s disease-like tau pathology *in vivo*^19^ and used to stratify participants for a recent clinical trial targeting amyloid pathology.^20^ However, at present, tau-PET is not widely accessible to patients compared to other neuroimaging methods.^21^ Moreover, the addition of tau-PET to the diagnostic evaluation of dementia, which currently includes ^18^F-fluorodeoxyglucose (FDG)-PET and amyloid-PET, creates an additional burden on patients of undergoing the test and the exposure to multiple radiopharmaceuticals. In addition, FDG-PET has wide application across all forms of degenerative dementia because it contains useful features across the entire spectrum of aetiologies beyond amyloid- and tau-associated conditions.^22^ Nevertheless, measuring tau pathology is integral to the diagnosis and prognosis of the Alzheimer’s disease continuum and increasing the accessibility of tau-PET has potential to enable a greater role in research and clinical applications in the future.^23^

In this study, using a large collection of multi-modality database (1192 unique individuals), we developed a convolutional neural network (CNN) model, which enables a cross-modal tau-PET synthesis from other neuroimaging data, including FDG-PET, structural T_1_-weighted MRI (T1w) or amyloid-PET, as input. Tau burden has been correlated to regions of FDG hypometabolism,^8,15,24,25^ cortical atrophy^10,13,26,27^ and amyloid accumulation,^26,28-30^ although the correlation values are limited given the complex relationship between the biomarkers. We hypothesized that the CNN model trained on a large neuroimaging sample might enable an accurate imputation of spatial distribution of tau pathology by learning the underlying biological relationship between biomarkers. This might be useful for a medical decision-making as the approach can provide a clinically useful mapping from one modality to another. With recent advances in deep learning techniques, several works have explored cross-modality synthesis that transforms images from one domain to another, including low-dose FDG-PET to standard-dose FDG-PET,^31^ CT to T1w,^32^ T1w to FDG-PET^33,34^ and CT to FDG-PET.^35^ In the current work, we present a 3D Dense-U-Net model for the imputation of tau-PET from either FDG-PET, amyloid-PET or structural T1w and compare the performance of each modality-based tau-PET imputation models. Moreover, we evaluate the clinical implications of the artificial intelligence (AI)-imputed tau-PET by assessing its predictive ability for classifying tau positivity and clinical diagnostic groups.

## Materials and methods

### Participants

Participants from the Mayo Clinic Study of Aging (MCSA) or the Alzheimer’s Disease Research Center study (ADRC) who underwent T1w, FDG-PET, amyloid-PET with ^11^C-PiB (Pittsburgh compound B)^36^ and tau-PET with ^18^F-Flortaucipir (AV-1451)^37^ were included (*n* = 1,192, number of scans = 1505) (Table 1 and Supplementary Fig. 1). All participants or designees provided written consent with the approval of Mayo Clinic and Olmsted Medical Center Institutional Review Boards. The inter-scan interval between input imaging and tau-PET was 6.53 ± 10.53, 6.50 ± 13.63 and 0.71 ± 5.58 days [mean ± standard deviation (SD)] for the FDG-PET, T1-MRI and PiB PET, respectively. The participants were categorized into major clinical subgroups based on clinical diagnosis including cognitively unimpaired (CU; *n* = 739, number of scans = 890), mild cognitive impairment (MCI; *n* = 169, number of scans = 208), typical Alzheimer’s disease (AD; *n* = 110, number of scans = 165), behavioural variant of frontotemporal dementia (bvFTD; *n* = 25 number of scans = 32), dementia with Lewy bodies (DLB; *n* = 38, number of scans = 54) and other clinical syndromes [e.g. vascular cognitive impairment, idiopathic REM sleep behaviour disorder (RBD), posterior cortical atrophy (PCA), semantic dementia, logopenic variant of primary progressive aphasia (lvPPA), non-fluent variant of primary progressive aphasia (nfvPPA) and progressive supranuclear palsy (PSP); *n* = 111, number of scans = 156] (Table 1). The clinical categories in these databases were not used in training the algorithm, given that ground-truth was the tau-PET scan from these participants. However, we evaluated the implications of the trained models using common clinical categories (cognitively unimpaired, Alzheimer’s disease spectrum, FTD spectrum and DLB spectrum).

**Table 1:** Demographics for Mayo participants

Characteristic	Clinical diagnosis					
	Normal	MCI	AD	FTD	DLB	Others
*n* (%)	739 (62)	169 (14.18)	110 (9.23)	25 (2.10)	38 (3.19)	111 (9.31)
Age, median (min, max), years	69 (30, 94)	74 (26, 98)	72 (53, 92)	62 (43, 75)	70 (45, 89)	68 (33, 85)
Male sex, *n* (%)	385 (52.10)	118 (69.82)	53 (48.18)	11 (44)	33 (86.84)	64 (57.66)
Education, median (IQR), years^a^	16 (13–17)	16 (12–18)	16 (13–17)	16 (13.75–18)	15.5 (14–18)	16 (14–18)
Clinical Dementia Rating Scale-Sum of Boxes, median (IQR)^b^	0 (0–0)	1 (0.5–1.5)	4.5 (2.5–7)	4.25 (2.75–6.25)	4.5 (3–6)	1.5 (0.5–4)
Meta-ROI FDG PET SUVR, median (IQR)	1.55 (1.46–1.64)	1.39 (1.28–1.49)	1.19 (1.05–1.30)	1.42 (1.32–1.55)	1.17 (1.08–1.27)	1.42 (1.22–1.56)
Meta-ROI PiB PET SUVR, median (IQR)^c^	1.39 (1.31–1.52)	1.55 (1.37–2.32)	2.49 (2.23–2.72)	1.34 (1.20–1.46)	1.61 (1.36–2.22)	1.43 (1.31–2.14)
Meta-ROI Tau PET SUVR, median (IQR)	1.18 (1.13–1.23)	1.24 (1.19–1.40)	1.91 (1.60–2.31)	1.25 (1.18–1.35)	1.21 (1.17–1.28)	1.26 (1.16–1.55)

AD = Alzheimer’s disease; DLB = dementia with Lewy bodies; FTD = frontotemporal dementia; IQR = interquartile range; MCI = mild cognitive impairment; ROI = region of interest.

^a^Number of participants missing this variable = 3.

^b^Number of participants missing this variable = 9.

^c^Number of participants missing this variable = 19.

To examine whether the trained model presents a dataset-specific bias, we also utilized the Alzheimer’s Disease Neuroimaging initiative (ADNI; adni.loni.usc.edu) dataset. For the ADNI cohort, we pulled all visits with a 3 T accelerated T1w, FDG-PET and tau-PET where available (*n* = 288; Supplementary Table 1). Amyloid-PET from the ADNI database was not used for the external evaluation because different amyloid tracers (Florbetapir^38^ and Florbetaben^39^) were used in that study. The ADNI dataset included normal controls (*n* = 15), MCI (*n* = 205) and dementia (*n* = 68). Image IDs for the ADNI cohort used in this study can be downloaded from the following link (https://github.com/Neurology-AI-Program/AI_imputed_tau_PET/ADNI_cohort_with_imageIDs.csv).

### Neuroimaging

For the Mayo data, T1w MRI scans were acquired using 3 T GE and Siemens scanners with MPRAGE sequences. PET images were acquired 30–40 min after injection of ^18^F-FDG, 40–60 min after ^11^C-PiB injection and 80–100 min after ^18^F-AV-1451 injection. CT was obtained for attenuation correction. Details of ADNI imaging protocols have been previously published.^40,41^ PET images were analysed with our in-house fully automated image processing pipeline.^42^ Briefly, the PET scans were co-registered to the corresponding T1w for each participant within each time point, and subsequently warped to template space^43^ using the non-linear registration. FDG-PET standardized uptake value ratio (SUVR) was calculated by dividing the median uptake in the pons and the cerebellar crus grey matter for tau-PET and PiB-PET SUVR.^44^ For each T1w volume, spatial inhomogeneities were corrected and an intensity normalization was performed by dividing a mean intensity derived from individualized white matter mask.^45^ The FDG, intensity-normalized T1w and PiB SUVR images were used for input data to the CNN model. The tau-PET SUVR images were used for target label data for CNN training. The PET images were not partial volume corrected. Cortical thickness and grey matter volume were measured with FreeSurfer software.^46^ The tau-PET meta-region of interest (ROI) used in this study included the amygdala, entorhinal cortex, fusiform, parahippocampal and inferior temporal and middle temporal gyri.^44,47^ The meta-ROI SUVR was calculated as an average of the median uptake across regions of meta-ROI. The diagnostic group-averaged images are displayed in Supplementary Fig. 2.

### Network architecture

**Figure 1:**
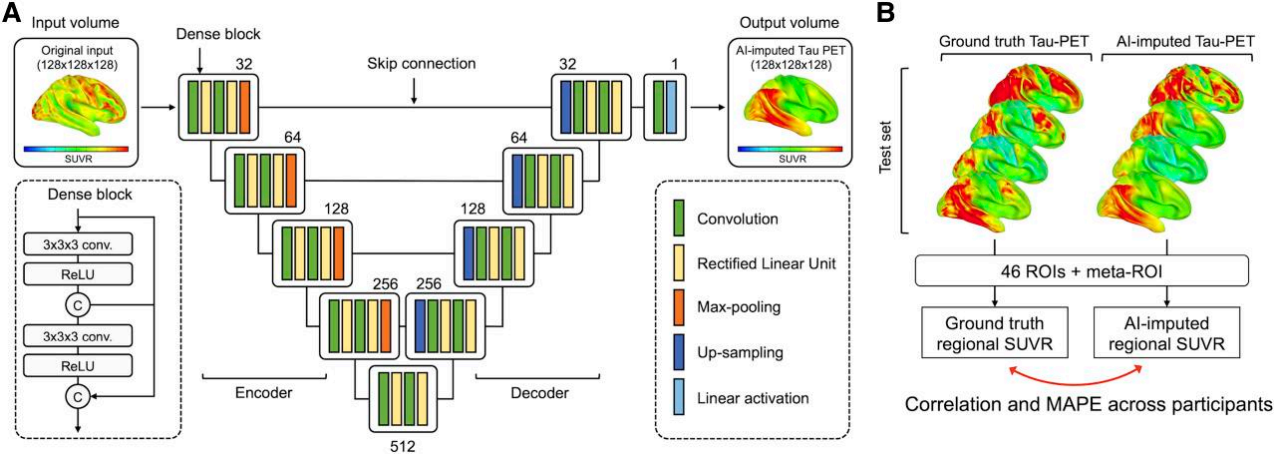
**Dense-U-Net architecture and layout of analysis**. (**A**) The architecture receives input of size 128 × 128 × 128 and produces the artificial intelligence (AI)-imputed tau-PET of the same dimension with input data. Dense-U-net architecture is composed of encoder (*left*), decoder (*right*) and bridge. *Left* dotted box illustrates a layout of dense connection in dense block, when output from each rectified linear unit (ReLU) layer is concatenated (circular C) to the input of the block before fed to the next layer. The numbers denoted above the dense blocks indicate a number of filters. (**B**) The similarity between ground-truth tau PET and AI-imputed tau PET was assessed across total participants in test set using a regional standardized uptake value ratio (SUVR) calculated from 46 regions of interest (ROIs) and meta-ROI. Pearson’s correlation and mean absolute percentage error (MAPE) were used as an evaluation metric.

A schematic of the 3D Dense-U-Net architecture used for this study is shown in Fig. 1.^48^ The network is a U-Net type architecture^49^ with dense interconnections between convolutional layers (dense block). The architecture is comprised of four downsampling (encoder) blocks for feature extraction and four upsampling (decoder) blocks for image reconstruction, which are connected by a bridge block. Within every block, the convolutional layers are densely interconnected in a feed-forward manner. The network doubles or halves the number of filters (denoted above each block) along each successive encoder and decoder path, respectively. The architecture takes input volumes of size 128 × 128 × 128 and outputs the images with the same dimensions. For this, we resized the volume by cropping and zero padding so that it is divisible by two until the bottom of the network for the max-pooling and upsampling procedure. Along the anterior-posterior axis, eight slices of anterior and nine slices of posterior were cropped. Then, seven slices were padded on the left and bottom of the cropped volume, forming a 3D data of size 128 × 128 × 128. The output volume of the network was reconstructed as the original size (121 × 145 × 121) for the visualization.

### Training and testing

The neural network was implemented using Keras with TensorFlow as the backend. Cross-validation experiments were conducted using 5-fold validations (60% training set, 20% validation set and 20% test set). To prevent any possible data leakage between the training and validation/testing datasets, we excluded any overlap of participants among training, validation and test sets. Within each set, multiple scans per subject were included. Demographics for participants in the training, validation and testing datasets for each fold can be found in Supplementary Tables 2 and 3, including pertinent clinical variables, measures of cognitive performance and tau-PET meta-ROI SUVR, which can be considered as a ground-truth measure of model training. As results, we found that the composition of each group was relatively similar. The model was optimized using Adam optimizer^50^ with parameters: β1 = 0:9 and β2 = 0.999. The training epoch used was 150. The learning rate from training was set to 1 × 10^−4^ and decreased by a factor of two for every 10 epochs. If the validation error did not improve in seven epochs, the learning rate was updated. A mini batch of size 2 was used. The mean squared error was used as the loss function.

### Occlusion analysis

Occlusion sensitivity analysis was performed to identify ROIs in the brain contributing to the performance of the tau-PET synthesis model.^51^ The analysis was conducted for the test image dataset for FDG-, T1w- and PiB-based models. For each model, voxels from a single ROI in the original source images were occluded with zero values one at a time, and their relevance in the tau-PET synthesis was estimated as a change of regional mean absolute percentage error (MAPE) between the original and after occlusion (ΔMAPE_R1→R2_ = MAPE_R1→R2__−_ MAPE_R2_, where R1 is an occluded ROI and R2 is a region where the MAPE is calculated).

### Neuropathology methods

The accuracy of AI-imputed tau-PET was evaluated using post-mortem neuropathology data. For the neuropathologic assessment, immunohistochemistry was performed using a phospho-specific tau antibody (AT8; 1:1000; Endogen).^5^ The AT8 immunostained sections were used to assess Braak tangle stage.^52,53^ Participants were assigned the neuropathologic diagnosis of Alzheimer’s disease if they had a Braak tangle stage of ≥IV and had at least a moderate neuritic plaque score. Primary age-related tauopathy (PART) was assigned if the case met published criteria—Braak tangle stage I–IV and Thal amyloid phase 2 or less. Lewy body disorders were classified neuropathologically based on the distribution and severity of Lewy bodies and neurites.^54^

### Statistical analysis

To evaluate the model’s performance, regional SUVRs were extracted from both the ground-truth and AI-imputed tau-PET scans and the Pearson’s correlation and MAPE between tau images across participants were tested. A difference of correlation coefficient and MAPE between the models was evaluated using a two-way ANOVA. Correlations between SUVR in the meta-ROI and Braak tangle stage were calculated using Spearman’s correlation. The tau positivity was defined using four different meta-ROI cut-off thresholds (1.11, 1.21, 1.33 and 1.46).^20,44^ Receiver operator characteristic (ROC) analyses were performed using the entire cohort; however, the T1w-related variables (i.e. cortical thickness and T1w-imputed tau) were analysed separately by their manufacturer (GE and Siemens) because combining the cortical thickness values across the manufacturer is not reliable. For each cut-off value, a pair-wise comparison of the area under the ROC curve (AUROC) was performed using a one-way ANOVA with Holm-Sidak *post hoc* test. The diagnostic groups were defined as CU amyloid negative (CUA−) and CU amyloid positive (CUA+), MCI, AD-spectrum [i.e. Alzheimer’s disease spectrum including typical Alzheimer’s disease, logopenic progressive aphasia (LPA) and PCA], FTD-spectrum (i.e. FTD spectrum including PSP, bvFTD, semantic dementia and nfvPPA) and DLB-spectrum (i.e. DLB spectrum including RBD and DLB) and the classification was performed for CU versus AD-spectrum, AD-spectrum versus FTD-spectrum, and AD-spectrum versus DLB-spectrum. A pair-wise comparison of the AUROC value for classifying diagnostic groups was performed using a two-way ANOVA and Holm-Sidak *post hoc* test. Beyond the performance evaluation, transparency in AI models is crucial for downstream users across various domains to determine whether a model is suitable for their use cases. To document the transparency of the trained model, we developed a model card accompanying benchmarked evaluation in a variety of conditions, such as across different race, ethnicity and demographics. The model card can be downloaded from the following link (https://github.com/Neurology-AI-Program/AI_imputed_tau_PET.git).

## Results

### Metabolic PET image-based tau-PET imputation

**Figure 2:**
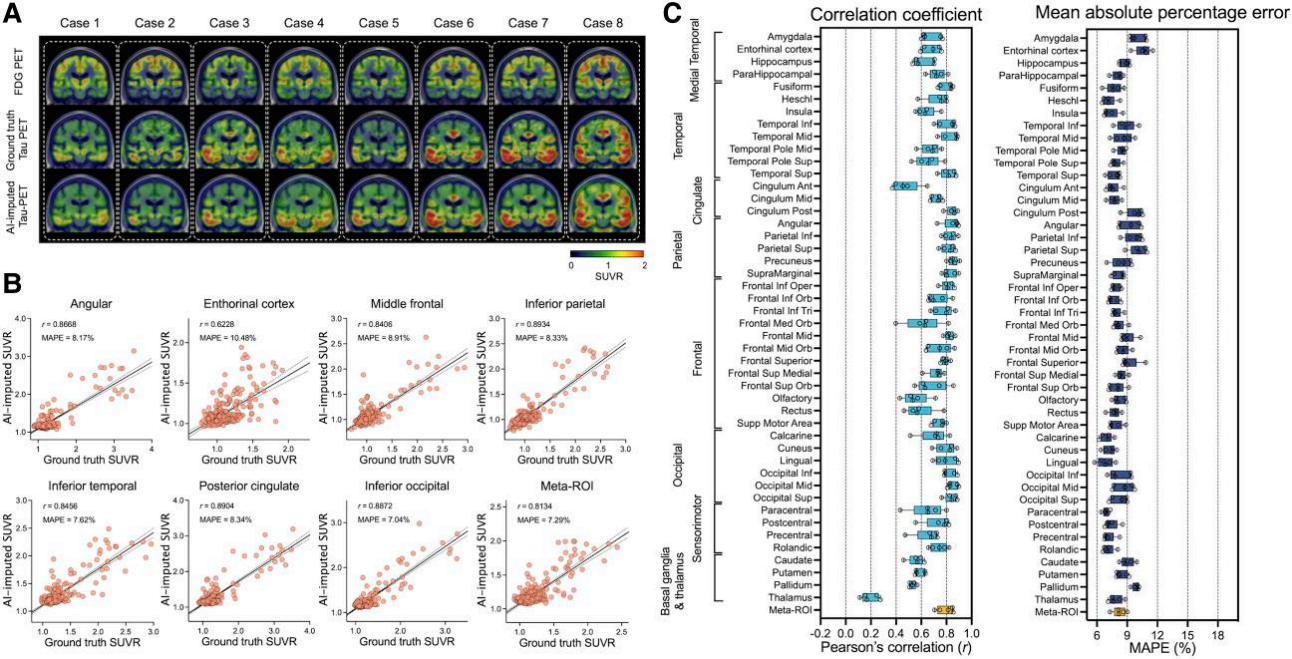
**FDG-PET based tau-PET synthesis results**. (**A**) Eight representative cases with original FDG-PET, ground-truth tau-PET and artificial intelligence (AI)-imputed tau PET. (**B**) Scatter plots of ground-truth tau-PET and AI-imputed tau PET from seven representative regions of interest (ROIs) and meta-ROI. *r* indicates Pearson’s correlation coefficient. MAPE = mean absolute percentage error. Linear regression (black line) and 95% confidence bands (dotted lines) are shown. (**C**) The mean of correlation coefficient and MAPE of five folds from 46 ROIs and the meta-ROI is summarized in a box plot. The yellow-coloured box depicts the meta-ROI result. Open circles indicate different folds. SUVR = standardized uptake value ratio.

First, we tried to impute tau-PET using glucose metabolism images obtained by FDG-PET. Figure 2A shows eight representative example cases from the test set, comparing the original FDG-PET, ground-truth tau-PET and AI-imputed tau-PET. As illustrated in Fig. 2A, the AI-imputed tau-PET image showed good agreement with ground-truth images in visual assessment. A high degree of similarity was observed for cases with high tau burden (Cases 6, 7 and 8) and cases with subtle tau tracer activity (Cases 1 and 2), demonstrating the range of tau activity the model is capable of characterizing.

To quantify the model’s performance, regional SUVRs and meta-ROI SUVRs extracted from both the ground-truth and AI-imputed tau-PET scans were compared (Fig. 1B). The AI-imputed tau-PET SUVR, when plotted against ground-truth tau-PET using a real tau tracer, demonstrated that each regional SUVR showed a high correlation (*r* > 0.8) and low MAPE (∼8%), which is moderately higher than the test-retest variability of AV-1451 PET,^55^ as well as the meta-ROI (Fig. 2B). The mean correlation coefficient and MAPE of 5-fold summarized for each anatomic ROI and the meta-ROI reflect the performance of the model (Fig. 2C). The mean correlation coefficient for the meta-ROI was 0.79 ± 0.06 and the MAPE was 8.24 ± 0.64%. The regional SUVR of the basal ganglia and thalamus, a known region of off-target AV-1451 binding, showed relatively lower performance.

To examine whether the trained model presents a dataset-specific bias, we evaluated the performance of the Mayo-trained models on the multi-site cross-modal data from the ADNI (*n* = 288; Supplementary Table 1). Using the ADNI scans, we observed that the FDG model trained on the Mayo dataset (Supplementary Fig. 3) showed a robust performance on the external evaluation, although the overall performance slightly decreased compared to the original result from the Mayo test set [*F*(1376) = 386.6, *P* < 0.001 for correlation coefficient and *F*(1376) = 1330, *P* < 0.001 for MAPE, using a two-way ANOVA].

### Tau PET imputation using structural MRI or amyloid-PET as input

**Figure 3:**
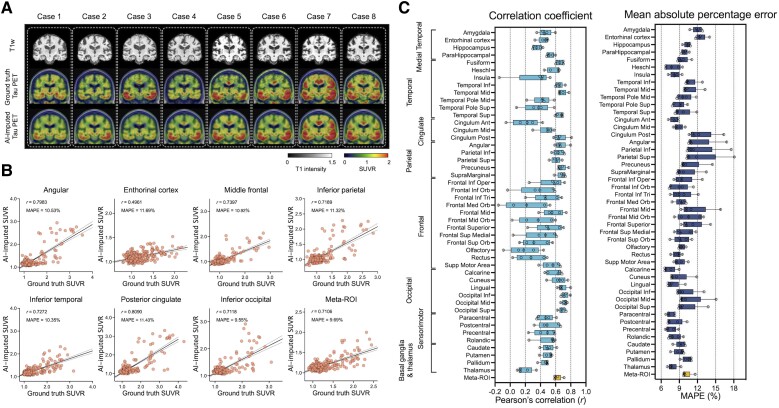
**Structural MRI-based tau-PET synthesis results**. (**A**) Eight representative cases with original T_1_-weighted (T1w), ground-truth tau-PET and artificial intelligence (AI)-imputed tau-PET. (**B**) Scatter plots between ground-truth tau-PET and AI-imputed tau-PET from seven representative regions of interest (ROIs) and meta-ROI. *r* indicates the Pearson’s correlation coefficient. MAPE = mean absolute percentage error. Linear regression (black line) and 95% confidence bands (dotted lines) are shown. (**C**) The mean correlation coefficient and MAPE of five folds from 46 ROIs and meta-ROI is summarized in the box plots. The yellow-coloured box depicts the meta-ROI result. Open circles indicate different folds. SUVR = standardized uptake value ratio.

Next, we used the same Dense-U-Net architecture to impute tau-PET using structural T1w. The model was initialized and separately trained from scratch. The prediction accuracy of T1w model was significantly lower than the FDG-based model [*F*(1376) = 424.1, *P* < 0.001 for correlation coefficient and *F*(1376) = 159.5, *P* < 0.001 for MAPE, using a two-way ANOVA; Fig. 3]. In some ROIs such as the insula, anterior cingulate, medial orbitofrontal, olfactory and gyrus rectus, the correlation coefficient was considerably low (*r* < 0.3; Fig. 3B and C). In the T1w-based model, the meta-ROI’s mean correlation coefficient was 0.62 ± 0.05 and mean MAPE was 10.16 ± 0.82% across the 5-fold test sets.

The T1w-based model was also cross-evaluated using the ADNI dataset. As a result, the performance of the T1w-based model on an external dataset was found to have relatively low accuracy compared to the training (Mayo) dataset [*F*(1376) = 134.1, *P* < 0.001 for correlation coefficient and *F*(1376) = 208.0, *P* < 0.001 for MAPE, using a two-way ANOVA; Supplementary Fig. 4]. In most ROIs, the correlation coefficient was very low (*r* < 0.2) and the MAPE was high (>10%), meaning that the T1w-based model trained on the training dataset did not show a robust performance for images acquired in a multi-site external dataset.

**Figure 4:**
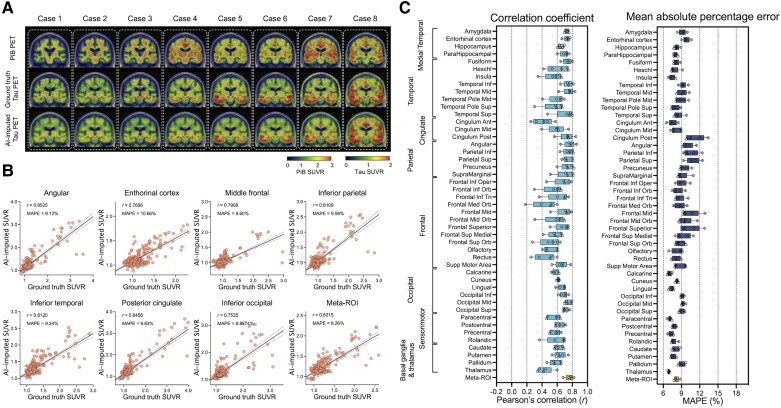
**Amyloid-PET based tau-PET synthesis results**. (**A**) Eight representative cases with actual Pittsburgh compound B (PiB)-PET, ground-truth tau-PET and artificial intelligence (AI)-imputed tau PET. (**B**) Scatter plots between ground-truth tau PET and AI-imputed tau PET from seven representative regions of interest (ROIs) and meta-ROI. *r* indicates the Pearson’s correlation coefficient. MAPE = mean absolute percentage error. Linear regression (black line) and 95% confidence bands (dotted lines) are shown. (**C**) The mean of correlation coefficient and MAPE of five folds from 46 ROIs and meta-ROI is summarized in a box plot. The yellow-coloured box depicts the meta-ROI result. Open circles indicate different folds. SUVR = standardized uptake value ratio.

Next, we trained the model using amyloid-PET inputs from the PiB radiotracer (Fig. 4). The PiB-based model was also able to generate AI-imputed tau-PET scans with high accuracy (Fig. 4A and B) and the mean correlation between ground-truth regional SUVR and AI-imputed regional SUVR was found to be 0.41–0.76 and the MAPE range was ∼7–11% (Fig. 4C). The general performance was significantly lower than the FDG-based model [*F*(1376) = 96.76, *P* < 0.001 for correlation coefficient and *F*(1376) = 30.77, *P* < 0.001 for MAPE, using a two-way ANOVA]; however, the performance was significantly higher than the T1w-based model [*F*(1376) = 137.7, *P* < 0.001 for correlation coefficient and *F*(1376) = 80.63, *P* < 0.001 for MAPE, using a two-way ANOVA]. The train and validation loss for each model are visualized in Supplementary Fig. 5.

### Further evaluation of the accuracy of artificial intelligence-based tau-PET imputation

For additional evaluation of the model’s performance, voxel-wise error maps between the AI-imputed tau and ground-truth tau-PET were calculated using a root mean squared error (RMSE) (Supplementary Fig. 6A–C). Overall, the FDG-based model showed the lowest RMSE across the cortical regions, followed by the PiB- and T1w-based models. For additional voxel-based quantitative analysis, multi-scale structural similarity index (MS-SSIM)^56^ was also computed (Supplementary Fig. 6D). All modalities showed moderately high MS-SSIM values (>0.9) and the performance of both the FDG- and PiB-based models was significantly higher than the T1w-based model (*P* < 0.001, Holm-Sidak test). Additional example images comparing ground-truth and AI-imputed tau-PETs are shown in Supplementary Fig. 7.

We then evaluated the accuracy of AI-imputed tau-PET using post-mortem neuropathology data by calculating correlations between SUVR in the meta-ROI and Braak tangle stage. Thirteen participants who had tau-PET within 3 years of death and complete neuropathologic assessments were eligible for evaluation. As a result, the FDG-PET SUVR was not significantly correlated with the Braak stage (*r* = −0.45, *P* = 0.126, Spearman’s correlation, Supplementary Fig. 8); however the AI-imputed tau-PET SUVRs from the FDG-PET showed a significant association with the Braak stage (*r* = 0.78, *P* = 0.003, Spearman’s correlation, Supplementary Fig. 8), which was also comparable with the ground-truth tau-PET (*P* > 0.05, *z*-test after Fisher’s *r* to *z* transformation, Supplementary Fig. 8). For the other modalities, the association was similar between the input imaging and AI-imputed tau-PET, or even worse than the input data for the T1w model.

We further explored combined modality training by simultaneously utilizing two input volumes (i.e. FDG-PET + T1w, FDG-PET + PiB PET and PiB PET + T1w) for tau-PET imputation (Supplementary Table 4). The architecture of the model remained unchanged, while adjustments were made to the input layer’s dimensions along the channel axis to take the superimposed bimodal volumes as inputs. As results, we found that FDG-based imputation outperformed other modalities significantly and employing a multimodal approach (e.g. FDG + PiB and FDG + T1w) did not enhance predictive accuracy compared to using solely FDG-PET as input. The multimodal training did not yield notable improvements for PiB-PET as well (PiB alone versus PiB + T1w).

In addition, we also trained different architectures: Variational Autoencoder (VAE)^57^ and a pix2pix Generative Adversarial Network (GAN),^58^ which have been reported for a paired image translation task. As a result, we found that both U-Net and pix2pix outperformed VAE across input modalities. Although the comparison between U-Net and pix2pix did not reach statistical significance, Dense-U-Net consistently showed higher performance compared to pix2pix (Supplementary Table 5 and Supplementary Fig. 9). In the within-architecture comparison, training with FDG- and PiB-based data consistently performed better across the models than with T1w (Supplementary Table 6). Based on these results, we speculate that because the input and output highly align with the same brain structure, the skip connection between the encoder and decoder included in the U-Net architecture is beneficial for improving imputation performance.

As a control experiment, we also trained a model to impute the PiB-PET from the FDG-PET. The results were rather suboptimal, showing a large deviation in the predicted PiB-PET SUVR compared to the ground-truth, yielding a high prediction error (Supplementary Fig. 10).

### Evaluation of artificial intelligence-imputed tau-PET’s clinical implications

Although the AI models, particularly the FDG- and PiB-based imputations, showed high accuracy in predicting the spatial distribution of tau pathology from input data, the clinical utility of AI-imputed tau images might be questionable due to the variability of predicted values. Therefore, we performed experiments to assess the clinical implications of AI-imputed tau-PET images. First, we performed ROC analyses for predicting tau positivity. In dementia research and clinical practice, although the biomarkers exist on a continuum, dichotomizing normal/abnormal tau using specific cut-points is useful and widely used.^44^ We tried to predict the tau positivity obtained from the ground-truth tau-PET data using four different meta-ROI cut-off thresholds (SUVR = 1.11, 1.21, 1.33 and 1.46) with the AI-imputed tau-PET. The lowest and highest cut-points (SUVR = 1.11 and 1.46) reflecting recent clinical trial stratification^20^ and middle cut-points reflecting 95% specificity (SUVR = 1.21) and discrimination between age-matched controls and cognitively impaired amyloid-PET positive individuals (SUVR = 1.33).^44^ In addition, to evaluate the performance of AI-imputed images relative to the input modalities, the ROC analysis was also performed using the actual FDG-PET, cortical thickness or PiB-PET as predictors. All variables were derived from the tau-PET meta-ROI for the analysis.

**Figure 5:**
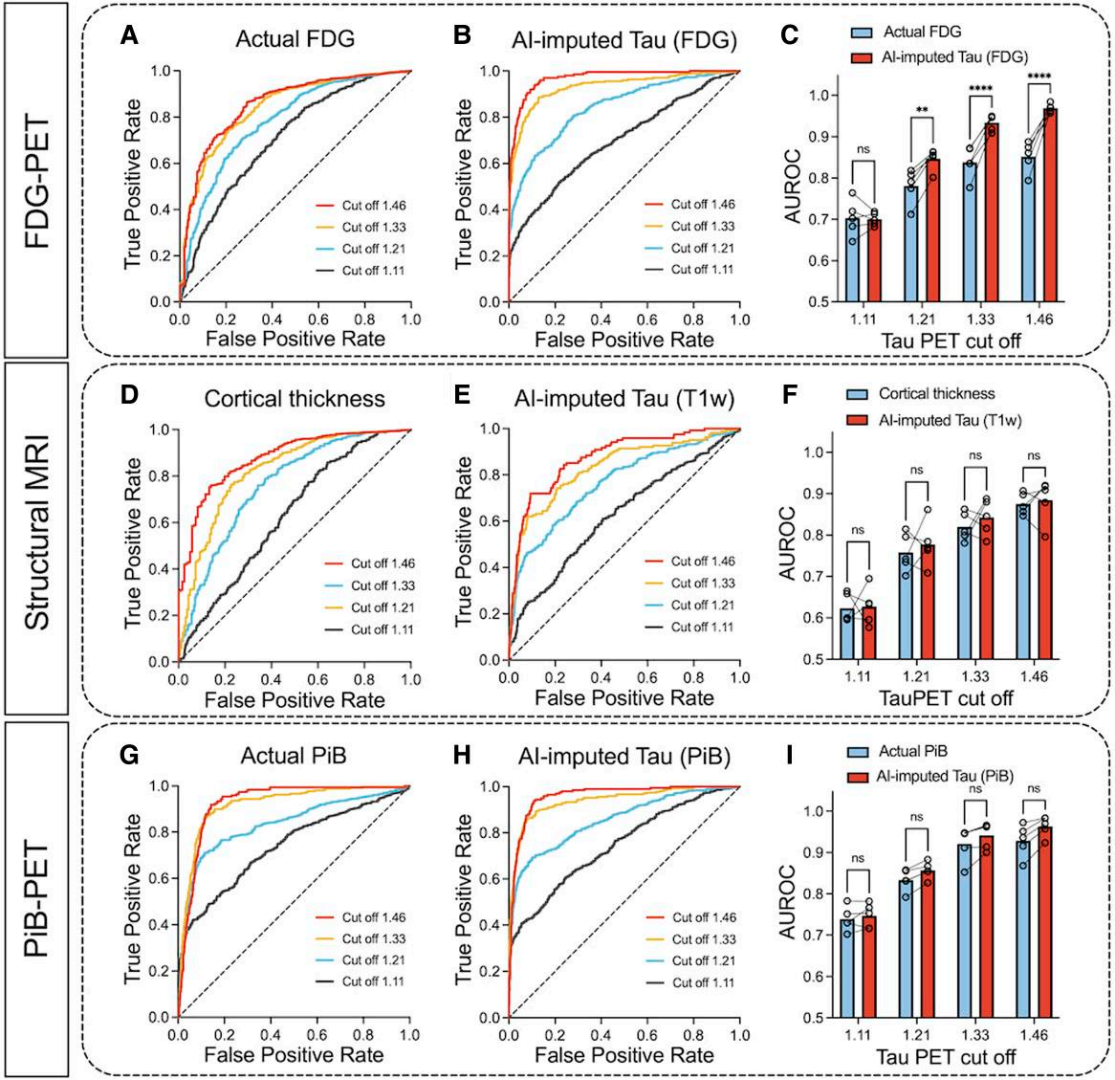
**ROC analysis for tau PET positivity**. Tau positivity predicted from the ground-truth tau-PET using four different meta-ROI (region of interest) cut-off thresholds (1.11, 1.21, 1.33 and 1.46) were obtained using six different predictors: (**A**) actual FDG-PET and (**B**) FDG-based synthesized tau-PET with (**C**) area under the ROC curve (AUROC) comparison between the original FDG and FDG-based AI-imputed tau-PET; (**D**) cortical thickness from the cohort who had GE scans and (**E**) T_1_-weighted (T1W)-based artificial intelligence (AI)-imputed tau-PET from the cohort who had GE scans with (**F**) AUROC comparison between the cortical thickness and T1W-based AI-imputed tau-PET. **(G**) Actual PiB-PET and (**H**) PiB-based AI-imputed tau-PET with (**I**) AUROC comparison between the PiB-PET and PiB-based AI-imputed tau-PET. A pair-wise comparison was performed between input data and the corresponding AI-imputed tau PET for each cut-off. Statistical significance was tested by *post hoc* Holm-Sidak comparisons after two-way ANOVA. **P < 0.005, ****P < 0.0001. Open circles in **C**, **F** and **I** indicate different folds. ns = not significant; ROC = receiver operating characteristic.

For FDG-PET, we found that applying the model was more successful in predicting tau positivity than the actual FDG SUVR (Fig. 5A–C). The FDG-imputed tau-PET showed significantly improved AUROC values versus the actual FDG, except at the lowest SUVR threshold (1.11) (*P* = 0.004 for 1.21, *P* < 0.001 for 1.33 and 1.46, Holm-Sidak test, Fig. 5C). A similar ROC analysis was performed using cortical thickness directly measured from T1w examinations and T1w-based AI-imputed tau-PET scans to predict true tau-positive participants (Fig. 5D–F). As the cortical thickness metric cannot be combined across the different manufacturers, the GE and Siemens cohorts were analysed separately and the result for GE, which was the majority manufacturer of our dataset, is displayed in the main result. In contrast to the FDG-based model, the T1w-based imputation was not more successful than direct measurement of cortical thickness (Fig. 5D–F). No significant differences were found in the AUROC (*P* > 0.05 for SUVR thresholds 1.11, 1.21, 1.33 and 1.46, Holm-Sidak test). The Siemens cohort showed a similar result (Supplementary Fig. 1^1^). The PiB-based model showed no significant improvement in the AUROC for tau prediction compared to actual PiB-PET SUVR (Fig. 5G–I). This result implies that imputing tau-PET scans from the FDG could augment clinical utility beyond using the FDG-PET alone. Meanwhile, the T1w and PiB model did not add predictive value for classifying tau positivity compared to the cortical thickness or PiB-PET SUVR. In the pairwise comparison between the predictors, FDG- and PiB-based AI-imputed tau-PET outperformed the other methods in classifying tau positivity, except for the lowest cut-off value (Holm-Sidak test, Supplementary Fig. 1^2^).

For the ADNI dataset, a similar result was observed (Supplementary Figs 13 and 14). FDG-based AI-imputed tau-PET showed significantly improved AUROC values over the actual FDG-PET (*P* < 0.001 for 1.11, 1.21, 1.33 and 1.46, Holm-Sidak test, Supplementary Fig. 13A–C), while the T1w-based model did not show an improved AUROC (*P* > 0.05, for SUVR thresholds 1.11, 1.21, 1.33 and 1.46, Holm-Sidak test, Supplementary Fig. 14D–F). For all SUVR thresholds, FDG-based AI-imputed tau-PET showed the highest AUROC value for classifying tau positivity.

**Figure 6:**
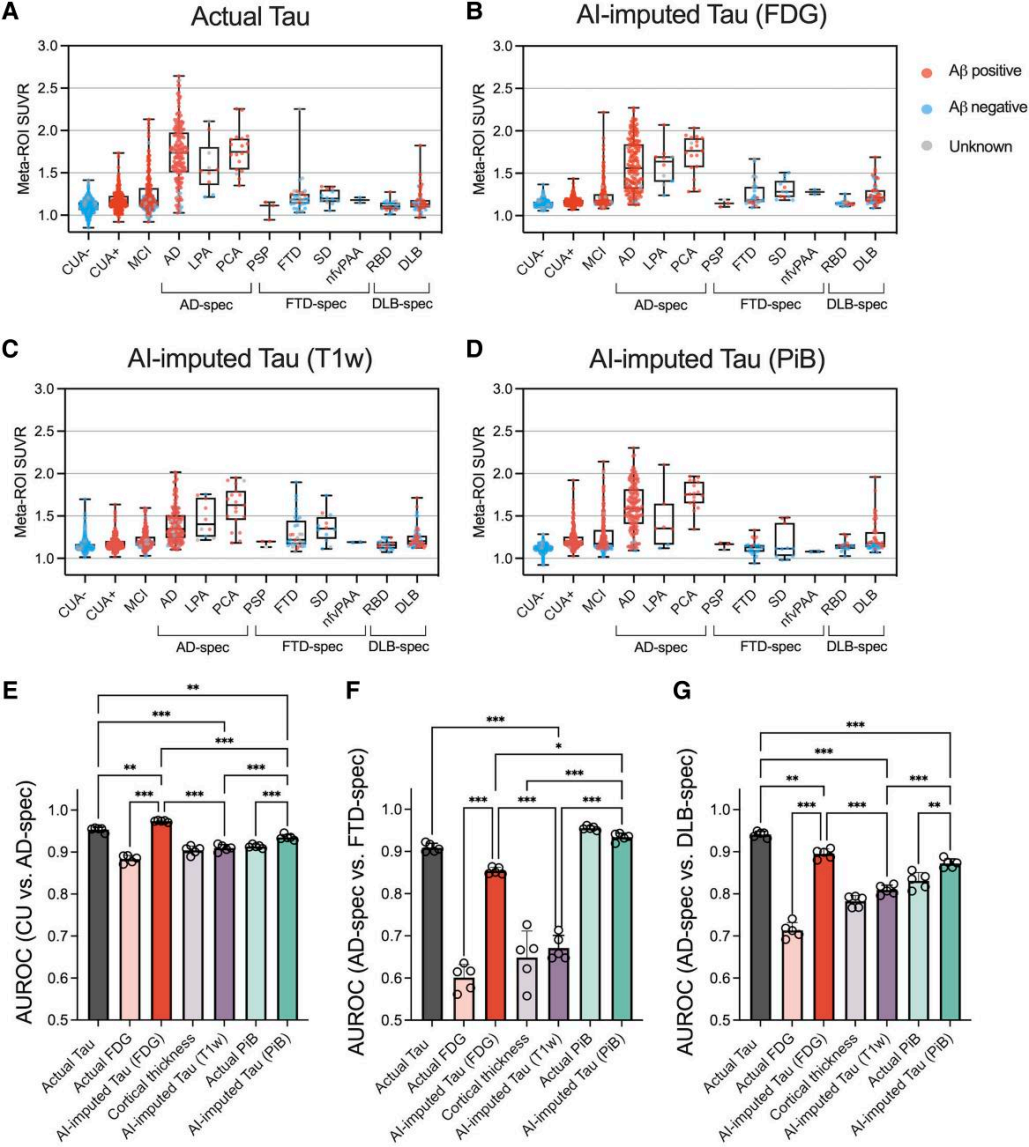
**Diagnostic performance of AI-imputed tau-PET**. (**A**–**D**) Meta-ROI (region of interest) standardized uptake value ratios (SUVRs) from the actual tau, FDG-, T_1_-weighted (T1W)-, and Pittsburgh compound B (PiB)-based artificial intelligence (AI)-imputed tau-PET were plotted for each diagnostic group. Red, blue and grey coloured dots show amyloid positive, negative and unknown, respectively. (**E**–**G**) Receiver operating characteristic (ROC) analysis was performed for classifying the diagnostic groups using seven predictors. Open circles indicate different folds. Statistical significance was assessed with two-way ANOVA and Holm-Sidak *post hoc* comparison. Aβ = amyloid-β; CUA = cognitively unimpaired with normal amyloid; CUA+ = cognitively unimpaired with abnormal amyloid level; MCI = mild cognitively impaired; AD = Alzheimer’s disease; LPA = logopenic progressive aphasia; PCA = posterior cortical atrophy; PSP = progressive supranuclear palsy; FTD = frontotemporal dementia; SD = semantic dementia; nfvPAA = non-fluent variant of progressive associative agnosia; RBD = REM sleep behaviour disorder; DLB = dementia with Lewy bodies; spec = spectrum. **P < 0.01, ***P < 0.0001.

For another experiment to evaluate the clinical implications of AI-imputed tau-PET, ROC analysis was performed to assess the diagnostic performance of AI-imputed tau-PET images. For this analysis, four different meta-ROI tau-PET values were extracted: actual tau, FDG-imputed tau, T1w-imputed tau and PiB-imputed tau (Fig. 6). For comparison with the model-imputed tau-PETs, metrics from each input modality were also calculated from tau-PET meta-ROI: FDG-PET SUVR, cortical thickness and PiB-PET SUVR. Figure 6A–D shows the meta-ROI tau-PET SUVR for each diagnostic subgroup. In addition, the scatter plots comparing the ground-truth versus AI-imputed SUVR has been visualized in Supplementary Fig. 1^5^. In general, the pattern of distribution was similar across the modalities, while the T1w-based tau-PET showed relatively lower predicted SUVR than others (Fig. 6C). The classification was performed for CU versus AD-spectrum, AD-spectrum versus FTD-spectrum and AD-spectrum versus DLB-spectrum (Fig. 6E–G). We performed a statistical test for comparisons of AUROC among the AI-imputed and actual tau-PET and pair-wise comparison between the AI-imputed tau-PET and the metric from the corresponding input data.

For the CU versus AD-spectrum comparison, the FDG-based model showed the highest accuracy followed by the actual, PiB-based and T1w-based tau-PET (Holm-Sidak test; Fig. 6E and H). Interestingly, the classification performance of the FDG-based model was significantly higher than the actual tau-PET (*P* < 0.001, Holm-Sidak test, Fig. 6E). In comparison with input modality, FDG- and PiB-based model showed improved accuracy (*P* < 0.001, Holm-Sidak test, Fig. 6E). For the AD-spectrum versus FTD-spectrum, the PiB-based model showed the best performance followed by the actual, FDG-based and T1W-based tau-PET (Fig. 6F); however, the PiB-PET also performed well and was not significantly different with the synthesized tau-PET (*P* = 0.65, Holm-Sidak test, Fig. 6F). The performance of FDG-based tau model was significantly improved compared to the FDG-PET (*P* < 0.001, Holm-Sidak test, Fig. 6F). In classifying the AD-spectrum versus DLB-spectrum, the actual tau performed the best, followed by the FDG-based, PiB-based and T1w-based AI-imputed tau-PET (Fig. 6G). FDG- and PiB-based model showed an improvement upon the performance of the input data (*P* < 0.001 and *P* = 0.007 for FDG-based model and PiB-based model, respectively, Holm-Sidak test, Fig. 6G).

The discriminatory performance of the models was further evaluated at different disease stages. First, we performed ROC tests to classify the diagnostic groups, specifically CUA− versus CUA+ and CUA+ versus cognitively impaired including the MCI and Alzheimer’s disease individuals (Supplementary Fig. 16A and B). For the CUA− versus CUA+, an earliest stage of disease progression, amyloid-PET-based approach including actual PiB-PET and PiB-based AI-imputed tau-PET showed the highest performance. This result aligns with expectations as the amyloid status was defined by the PiB-PET level and tau-PET is known to be less sensitive for early disease stages. Interestingly, applying the model on the PiB-PET significantly decreased the prediction performance, which implies that converting PiB-PET to tau-PET by AI might result in some information loss. For the CUA+ versus cognitively impaired, the classification performance of AI-imputed tau-PETs was comparable with the actual tau-PET. Notably, only the FDG-based model was able to significantly improve the performance than that of the input modality. Additionally, we performed ROC tests for predicting tau positivity separately for cognitively unimpaired and cognitively impaired groups (Supplementary Fig. 16C and D). As a result, although the overall performance was low across all modalities in the CU group, the FDG- and PiB-based model showed high predictability (mean AUROC > 0.8) for the cognitively impaired group, with only FDG-based model surpassing the input data to predict tau positivity.

### Interpretability of 3D Dense-U-Net model using occlusion sensitivity analysis

**Figure 7:**
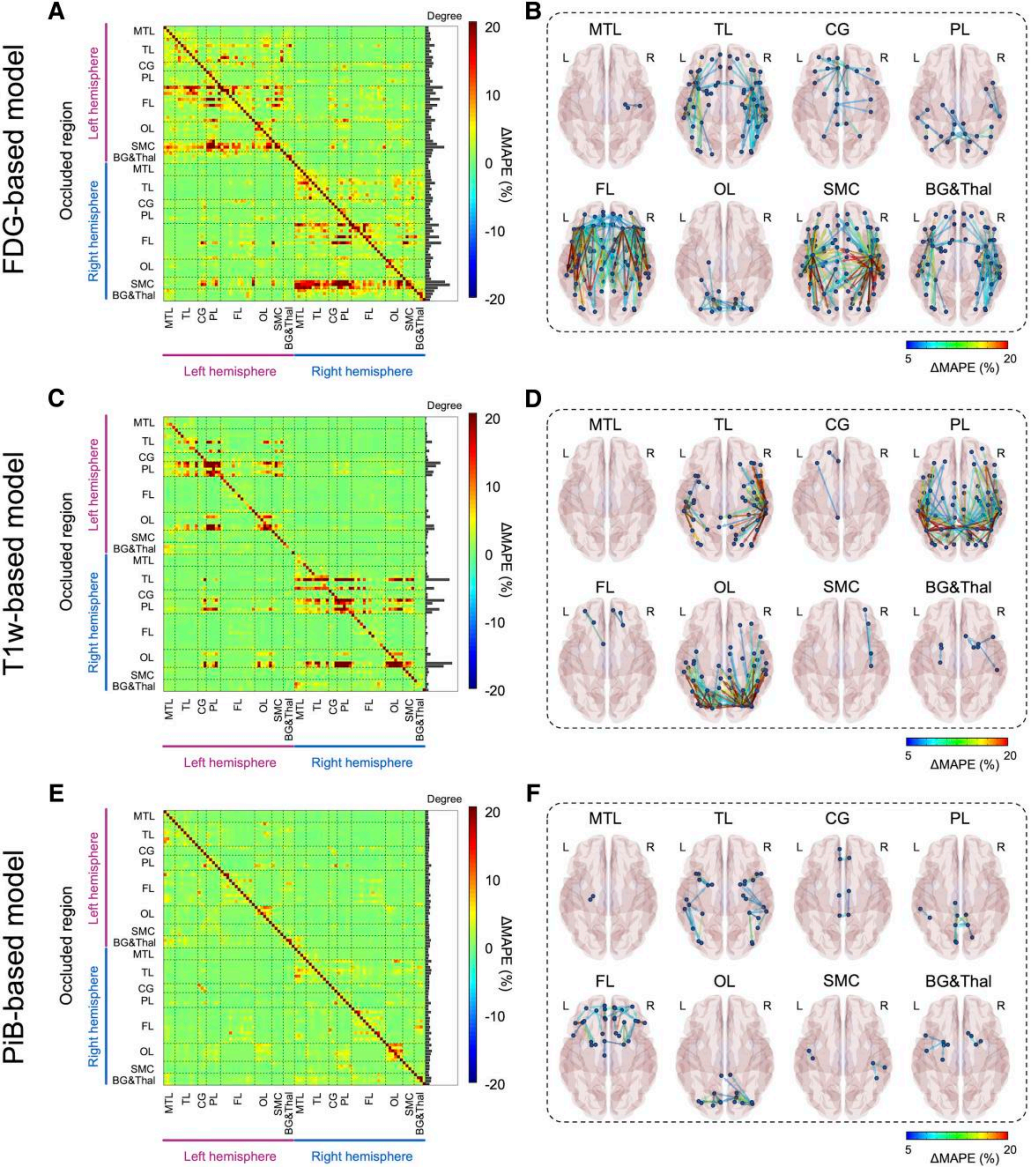
**Occlusion analysis**. Region of interest (ROI)-wise occlusion analysis was performed to enhance the interpretability of model. (**A, C** and **E**) The adjacency matrix shows the ΔMAPE (mean absolute percentage error) in one ROI (horizontal axis) from occluding another ROI (vertical axis) for FDG-, T1W- and PiB-based model, respectively. ΔMAPE was calculated as MAPE_R1→R2_ − MAPE_R2_, where R1 is an occluded ROI and R2 is the region where the MAPE is calculated. The *right* panel in each matrix indicates the summation of ΔMAPE along the horizontal axis. (**B, D** and **F**) 3D rendering plots of the adjacency matrix in **A**, **C** and **E** for FDG-, T1W- and PiB-based model, respectively. Each edge’s colour was illustrated by ΔMAPE value between nodes. Each label denoted above the figure indicate the occluded regions. BG&Thal = basal ganglia and thalamus; CG = cingulate cortex; FL = frontal lobe; MTL = medial temporal lobe; OL = occipital lobe; PL = parietal lobe; SMC = sensorimotor cortex; TL = temporal lobe.

To facilitate the interpretability of the Dense-U-Net model, saliency maps were estimated through occlusion sensitivity analysis for three different input modalities.^51,59^ In the occlusion method, a single ROI in the input space was occluded by setting these voxels to zero, and their relevance in the decisions was indirectly estimated by calculating the change of MAPE (i.e. ΔMAPE = MAPE_occlusion__−_ MAPE_original_). An adjacency matrix (Fig. 7A, C and E) plotting the regional ΔMAPE against each occluded ROI (vertical axis) shows the contribution of each ROI to the performance of the model. The diagonal line in each of these adjacency matrices is somewhat expected, representing the high contribution of the voxel of source images to the same region of the synthesized tau, observed for all three modalities (Fig. 7A, C and E). Notably, occlusion analysis revealed multiple additional anatomic regions with a high contribution that are spatially remote, which differ according to the input images. For the FDG-based model, the sensorimotor cortex and the frontal lobe were dominant contributors to the global accuracy of the tau model, showing a high contribution to the MAPE for most of the brain (Fig. 7A and B). This implies that metabolism in the sensorimotor cortex and the frontal regions were involved in the accurate imputation of tau-PET for other brain regions. On the other hand, for the T1w-based model, the temporal, parietal and occipital lobes were found to be the dominant contributor to global accuracy (Fig. 7C and D). The influence of remote structures was less prominently observed in the PiB-based model (Fig. 7E and F), implying that this model generates the AI-imputed tau-PET images using only relatively local amyloid information. For every model tested, no interhemispheric effect was observed.

## Discussion

We have described a new approach for cross-modality tau-PET image imputation using 3D Dense-U-Net models. Overall, FDG-based approaches showed the highest degree of accuracy with good correlation to ground-truth tau-PET and low error for regional SUVRs, followed by the PiB-based model. The performance of the T1w-based model was significantly inferior to the FDG- and PiB-based models. In addition, the FDG-based model showed the most robust prediction capability, performing accurately in an external cohort from the ADNI database where the T1w-model did not. In testing the clinically relevant application of AI-imputed tau-PET to predict tau positivity and classify diagnostic groups, only the FDG-based model showed significant improvement upon the performance of the original input data, suggesting that the model may enhance the utility of the metabolic images alone. The occlusion method, employed in an attempt to allow interpretation of the model’s mechanism of prediction, revealed that the FDG- and T1w-based models utilized global input from physically remote ROIs to impute the tau-PET, whereas a relatively locoregional contribution was predominantly observed in the PiB model.

We speculate that the Dense-U-Net models generated tau-PET images using the patterns of hypometabolism, cerebral atrophy and amyloid burden captured by FDG-PET, structural T1w MRI and PiB-PET, respectively. The possibility that FDG hypometabolism, atrophy and amyloid levels are important features of the model, facilitating the successful imputation of tau-PET images, is biologically plausible and supported by previous literature (see Supplementary Fig. 1^7^ for the associations between the modalities). A strong correlation of the tau uptake on tau-PET and hypometabolism on FDG-PET is well documented in prior studies.^8,24,60,61^ The regional atrophy pattern identified on T1w correlates well with regional tau-PET uptake.^10,13,26,27,62^ Autopsy studies also support a strong correlation of tau burden and brain atrophy.^63-66^ A correlation between tau and amyloid distribution has been shown, although the molecular relationship is complex, with a stronger relationship observed in the temporoparietal regions to a greater degree for predominantly cognitively normal cohorts^26,28-30^ and in the frontal, parietal and occipital lobes in a more advanced dementia cohort.^67^ These neuroimaging Alzheimer’s disease biomarkers become abnormal in a temporally ordered manner.^68^ The amyloid-PET tracer uptake increases earliest followed by tau-PET and FDG-PET, then structural MRI and finally clinical symptoms. The amyloid cascade hypothesis suggested that accumulation of Aβ plaques is the primary cause of tau NFT formation^69^; however, it has also been suggested that the aggregation of toxic form of Aβ and tau might be independent processes separately contributing to the development of Alzheimer’s disease pathology.^70^ In addition, autopsy data have shown that the regional patterns of Aβ differ from that of tau deposition.^71^ Meanwhile, hypometabolism and atrophy are more closely related to tau accumulation as a downstream consequence of neuronal loss due to tau NFTs. Abnormalities on FDG-PET may occur before structural changes in the brain in Alzheimer’s disease^72,73^ and hence potentially closer in time to the tau deposition, perhaps relating to the better performance of the FDG-based imputation in our study. Whitwell *et al*.^60^ also showed that FDG hypometabolism correlated with tau-PET uptake better than cortical thickness or PiB in both typical Alzheimer’s disease and atypical Alzheimer’s disease, implying that FDG metabolism is most sensitive to the effects of tau pathology. This is concordant with our observation that the FDG-based model was more successful than the T1w- and PiB-based model in comparison to ground-truth tau-PET. FDG has also been proposed as a marker of other conditions of interest in clinical dementia populations beyond those associated with Alzheimer’s disease-tau, such as hippocampal sclerosis and TDP-43,^74^ DLB^54,75,76^ and is currently approved and covered by Medicare for clinical use in the differential diagnosis of FTD and Alzheimer’s disease. This wide applicability across the spectrum of clinical conditions included in building our model is likely a key contributing factor in our model’s potential to enhance the clinical utility of FDG.

Furthermore, occlusion analysis revealed that the model’s predictions of tau activity for a given ROI depends not only on that same anatomic ROI but also on global input from physically remote ROIs. This held true for both the FDG- and T1w-based model’s predictions, whereas the PiB-based model demonstrated predominantly locoregional contributions to the tau prediction for each ROI. The result implies that local amyloid levels alone may be sufficient for the PiB-model to generate tau images while the trained model did not simply translate local FDG-PET activity or T1w features to tau-PET but relied on additional associations to inform the distribution of tau in the model-imputed PET images. This also provides clues about the enhanced performance of the FDG-based model compared to the actual FDG in contrast to the other models did not.

For the FDG-based model, one possible interpretation is that the model could predict the tau level based on SUVR comparisons between ROIs. The primary sensorimotor cortex and frontal lobe were dominant areas of influence on synthesized tau-PET accuracy from the FDG-based model revealed by occlusion analysis, surprising because the sensorimotor cortex is typically spared from FDG hypometabolism and frontal lobe involved in later stages of Alzheimer’s dementia.^77^ Therefore, preserved metabolism in these regions may be interpreted by the model as reference region, modifying the model’s prediction of tau uptake in remote locations of the brain. In this context, it is important to note that sparing of the sensorimotor strip in Alzheimer’s disease is a feature currently used by clinicians to inform expert visual interpretation of FDG-PET images.^78^ This clinically used feature is thought to be related to Alzheimer’s disease biology rather than image intensity normalization. This feature, juxtaposed with heteromodal association cortex, also characterizes principal patterns of functional connectivity^79^ that are also observed in modes of variation in FDG-PET related to global functional architecture across dementia syndromes.^22^ Therefore, one biologically plausible alternative explanation is that the model could utilize information related to the brain’s global functional architecture in predicting the local tau uptake. Previous studies have supported the relationship between tau pathology and brain connectivity, based on the study of tau-PET distribution and correlation to resting state functional MRI (fMRI).^9,14,80-82^ Franzmeier *et al*.^83^ also reported that the higher functional connectivity observed by the resting state fMRI is associated with higher rates of tau accumulation. However, different functional properties have been associated with amyloid,^82^ which may potentially explain the difference in learned features for these two modalities in predicting tau pathology in our study. This is consistent with our recent study showing that the FDG-PET based global functional state space showed a much higher predictive accuracy for tau-PET and Braak NFT stage than amyloid-PET.^22^ A key feature of the global functional state space described in that study is the juxtaposition of heteromodal association cortex with primary sensorimotor cortex, and this feature is reflected in FDG occlusion analysis (Fig. 7A and B), but not in the amyloid occlusion analysis (Fig. 7E and F) observed in the current study.

For the T1w-based model, the dominant regions of influence on remote parts of the brain were in the temporal, parietal and occipital lobes, correlating to areas of characteristic tau deposition and areas of significant regional volume loss in the Alzheimer’s disease spectrum.^4^ Similar to the FDG-based model, the T1w model utilized the global information to predict the tau level; however, applying the model did not improve the performance compared to cortical thickness. The marginal predictive accuracy of the T1w model, presumably hampered by the heterogeneity of structural changes, may not be robust enough to improve upon the structural images, which have higher spatial resolution compared to the PET scans. Structural imaging is also less sensitive to changes in functional networks than FDG-PET and therefore potentially less likely to contain the same level of functional network information on a single subject level that could be used to predict tau-PET.

Off-target binding of the tau tracer AV-1451 is an incompletely characterized phenomenon, most frequently described in the substantia nigra, caudate, putamen and choroid plexus on the basis of post-mortem analysis and autoradiography studies.^84-86^ The literature on the topic has suggested non-specific binding to structurally similar molecules such as MAO-A, MAO-B and potentially to mineralized or pigment-containing structures, such as neuromelanin. Because the off-target binding of AV-1451 is not correlated to hypometabolism, atrophy and Aβ burden, we would expect the off-target binding to be somewhat poorly predicted in the synthetic tau images and this is what we observed. The common locations of off-target binding that were included in the ROI analysis demonstrate relatively low correlation and high MAPE with the imputed tau-PET scans and ground-truth for all of the models (Figs 2–4). The basal ganglia regions showed some association between ground-truth and AI-imputed SUVR (Supplementary Fig. 18). Previous studies have shown that the non-specific binding in the basal ganglia is associated with age, as the neuromelanin and iron increases with age.^86,87^ Thus, we speculate that the AI model may impute the off-target binding by learning age-related changes of input.

The AI-imputed tau-PET is also limited, to a certain extent, by the properties of true ^18^F-Flortaucipir PET. The model follows the behaviour of AV-1451 PET, used as the ground-truth, and not necessarily the distribution of tau that might be found at autopsy. This is also a strength, in that the model generates a result that is analogous to a clinically useful diagnostic procedure; however, this also has some complex implications. The AV-1451 tracer varies in strength of binding to tau isoforms, binding less to 3R or 4R tau than 3R + 4R tau.^86^ This is also reflected *in vivo* suggesting more specificity of AV-1451 for Alzheimer’s disease-like tau than other tau isoforms.^19^ Numerous studies have confirmed that the role of AV-1451 in detecting non-Alzheimer’s tauopathies is limited.^88,89^ Interestingly, the FDG-based model’s overall prediction error was slightly higher in the temporal region for the FTD cohort and parietal region for the DLB cohort than the Alzheimer’s disease cohort (Supplementary Fig. 19), which are the regions with characteristic hypometabolism in each disease.^78,90^ This may in part reflect a less direct relationship between areas impacted by these isoforms, changes of FDG-PET and the predicted tau-PET activity. Nonetheless, the diagnostic performance of the model’s meta-ROI for these groups of FTD and DLB participants was generally accurate and significantly enhanced the performance of FDG-PET alone. The differences between the input and output of the model and the regional variation with different types of tau-pathology support our speculation that the model is not directly ‘translating’ FDG uptake into tau for specific region but is more likely utilizing global input from physically remote ROIs and broader pattern recognition mechanisms to predict tau activity for a given region.

The AI-imputed tau-PET may allow clinicians and researchers to maximize the use of neuroimaging biomarkers with a projection of tau pathology. Particularly, based on the experiments testing clinical implications of the synthesized tau-PET, application of the model would be most beneficial for FDG-PET as it could augment the utility of the metabolic images. The high correlation to ground-truth, including in an external dataset, implies that AI-imputed tau-PET may be a viable alternative of tau-PET in scenarios where tau-PET is not feasible, or the tau radiotracer is unavailable. As outlined in the ‘Introduction’, use of multiple radiopharmaceuticals for FDG, tau and amyloid-PET is an expensive and resource-intensive prospect, now emerging as an area of research and clinical need with recent FDA accelerated approval of Alzheimer’s disease-modifying therapy^91^ and the potential for additional targeted therapies in the future. In contrast to tau-PET agents, ^18^F-FDG-PET is one of the most widely available and utilized nuclear imaging modalities in current clinical practice.^92,93^ FDG-PET has the support of multiple professional societies in the diagnosis of dementia and is accessible at many medical centres.^94-100^ We hope that our proposed model could help maximize the clinical utility of the FDG-PET. Our study demonstrates that FDG-imputed tau-PET may provide valuable information regarding tau pathology with a high correlation to real tau-PET, especially in symptomatic individuals. Our work demonstrates the potential for the clinical utility of FDG-PET to be extended beyond differential diagnosis of FTD-spectrum, AD-spectrum and the DLB-spectrum to additional utility in stratifying patients on the AD-spectrum by tau load to facilitate complex clinical decision-making. This study also suggests potential applications in settings where only FDG-PET is feasible, and potential for use as a biomarker in the research setting. This opens the door to conduct further feasibility and real-world performance studies of the clinical utility of FDG-PET as the primary initial screening PET modality in a streamlined evaluation of patients with dementia symptoms of any isolated or mixed aetiology from a pathologic perspective. Such studies would require careful designs evaluating multiple biomarker strategies with real-world clinical outcomes that are beyond the scope of the current work. Ultimately, AI-imputed tau-PET may enable more efficient resource utilization and reduce patients’ exposure to multiple radiopharmaceuticals and imaging tests by maximizing the information gleaned from FDG-PET.

One important limitation of the study is that the PiB-based model was not cross-evaluated in the external dataset, as amyloid-PET using the PiB radiotracer was not available from the ADNI database. While the FDG model showed relatively robust performance during external validation, a significant decrease in performance was observed. Transfer learning could be a possible way to perform fine-tuning; however, we were unable to do so due to the insufficient sample size of the publicly usable database. The Mayo Clinic dataset used for training and evaluation in this study was large but predominantly cognitively unimpaired individuals where little and/or confined tau-PET uptake can be expected. Incorporating more samples with higher tau-PET levels can further enhance the model’s performance by enabling it to learn a wider range of patterns regarding the underlying biological relationship between the input modality and NFT accumulation. While the occlusion sensitivity analysis provides some insight regarding which regions are important to the success of the model, the mechanism of projecting tau uptake by the model remains unknown, hindering our ability to make inferences about the relationships between brain structural changes, tau and amyloid deposition, and glucose metabolism. We use the information here to imply relationships between tau deposition and metabolism in other parts of the brain, which merit exploration with further research. We acknowledge that these measurements include small regions, where measurements from PET images are nosier than in larger regions, but this limitation is common to most published analyses of tau-PET. The limited detectability of AV-1451 PET to early tau NFT accumulation^5,101^ as well as a variable resilience to tau pathology^102^ might penalize the reliability of the model’s prediction.

In summary, a 3D Dense-U-Net architecture is presented, which produced synthesized tau-PET brain scans from FDG-PET, PiB-PET and T1w. The FDG-based model of AI-imputed tau-PET demonstrated a high degree of correlation to ground-truth tau-PET for patients on the MCI-AD spectrum, distinguished tau-positive versus tau-negative patients, and classified diagnostic groups with performance similar to the AV-1451 tau-PET exams. AI-imputed tau is feasible and has a potential to augment the value of FDG-PET for MCI and Alzheimer’s disease patients. Although the bimodal training did not yield significant improvement, more thorough optimization of the model’s architecture to maximize the efficiency of multimodal inputs could potentially enhance the performance.

## Supplementary Material

awad346_Supplementary_Data

## Data Availability

In accordance with current standards for protection of sensitive patient information in the MCSA and ADRC that undergo continuous review by study leadership, the current data access policy indicates that qualified academic and industry researchers can request data, biospecimens or both (https://www.mayo.edu/research/centers-programs/alzheimers-disease-research-center/data-requests). Qualified researchers interested in these data sharing opportunities can submit a request to the centre’s executive committee. Once a request is submitted, the committee sends the indicated principal investigator an email confirming that the request was received and giving a timeline for committee review. Images from the Mayo ADRC that meet Standardized Centralized Alzheimer’s and Related Dementias Neuroimaging (SCAN) imaging criteria are available here (https://scan.naccdata.org/). Data are available from the National Alzheimer’s Coordinating Center (https://naccdata.org/requesting-data/data-request-process). Data from the Alzheimer’s disease Neuroimaging Initiative (ADNI) are available from the ADNI database (https://adni.loni.usc.edu/) upon registration and compliance with the data usage agreement. The source code is available online: https://github.com/Neurology-AI-Program/AI_imputed_tau_PET.
