# Supplementary material for: Synthesizing images of tau pathology from cross-modal neuroimaging using deep learning
Source: Brain. 2023 Oct 7;147(3):980–95. doi: 10.1093/brain/awad346 (PMC10907092; doi:10.1093/brain/awad346)
Supplement: awad346_Supplementary_Data [file awad346_supplementary_data.zip › brain-2023-00368-File010.pdf]

Supplementary Materials

Race and ethnicity of the dataset

Reporting race and ethnicity in this study is consistent with the Inclusion of Women, Minorities, and Children policy of the US National Institutes of Health (NIH). Participants were categorized based on the NIH Policy on Reporting Race and Ethnicity Data. The Mayo cohort included participants who self-reported as Asian, non-Hispanic (n=8, 0.53%), Black or African American, non-Hispanic (n=10, 0.66%), Hispanic or Latino (n=63, 4.19%), Native Hawaiian or Other Pacific Islander, non-Hispanic (n=1, 0.07%), White, non-Hispanic (n=1,407, 93.49%), Choose Not to Disclose (n=5, 0.33%), other (n=1, 0.07%), and unknown (n=10, 0.66%). The ADNI cohort included participants who self-reported as Asian, non-Hispanic (n=3, 1.04%), Black or African American, non-Hispanic (n=16, 3.47%), Hispanic or Latino (n=16, 3.47%), White, non-Hispanic (n=248, 86.11%), Multiracial, non-Hispanic (n=4, 1.39%) and unknown (n=10, 0.66%).

Supplementary Figures

Supplementary Fig.1. Data inclusion/exclusion criteria.

Supplementary Fig.2. Diagnostic group-averaged images.

Supplementary Fig.3. Testing the FDG-based model on the ADNI dataset.

Supplementary Fig.4. Testing the T1W-based model on the ADNI dataset.

Supplementary Fig.5. Train and validation loss as the epoch increases.

Supplementary Fig.6. Voxel-wise error maps and multi-scale structural similarity index.

Supplementary Fig.7. Comparisons of performance among the AI-imputed tau PET and ground truth tau PET in 3-dimensional stereotactic surface projection images.

Supplementary Fig.8. AI-imputed tau PET and Braak stage.

Supplementary Fig.9. Regional correlation coefficient from the different architectures.

Supplementary Fig.10. FDG PET based PiB PET synthesis results.

Supplementary Fig.11. ROC analysis for the Siemens cohort.

Supplementary Fig.12. AUROC comparisons for tau positivity.

Supplementary Fig.13. ROC curves showing the classification performance on the tau positivity tested on ADNI dataset.

Supplementary Fig.14. AUROC comparisons for different MRI manufacturers tested on ADNI dataset.

Supplementary Fig.15. Disease group specific scatter plots between the ground truth SUVR vs. AI-imputed SUVR.

Supplementary Fig.16. Evaluation of the model's performance at different disease stages.

Supplementary Fig.17. Associations between the input modality and tau PET meta-ROI SUVR.

Supplementary Fig.18. AI-imputed SUVR of regions of off-target bindings.

Supplementary Fig.19. Regional MAPE distribution for FDG-based AI-imputed tau-PET for AD, FTD, and DLB diagnostic groups.

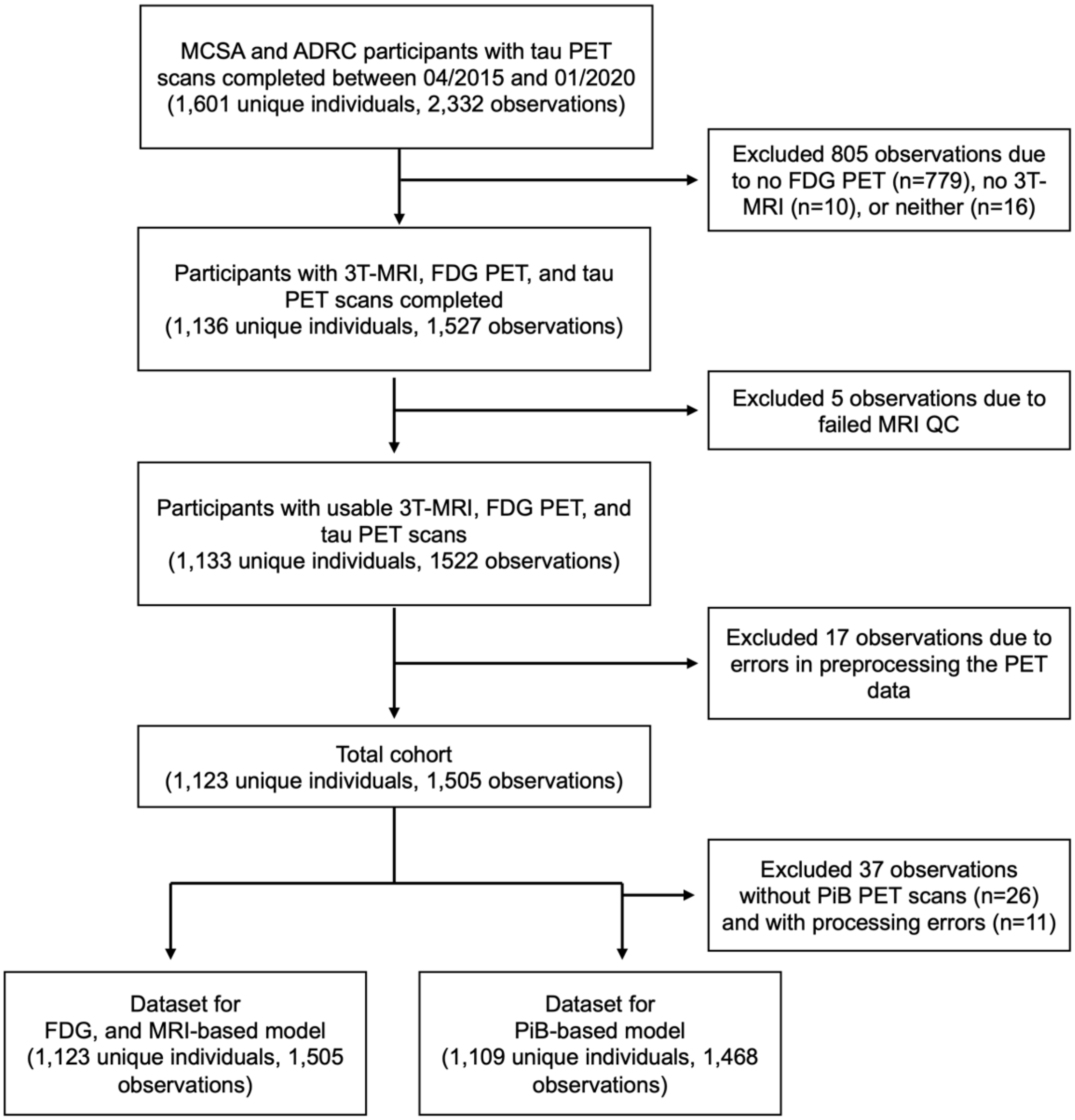

Supplementary figure 1. Data inclusion/exclusion criteria.

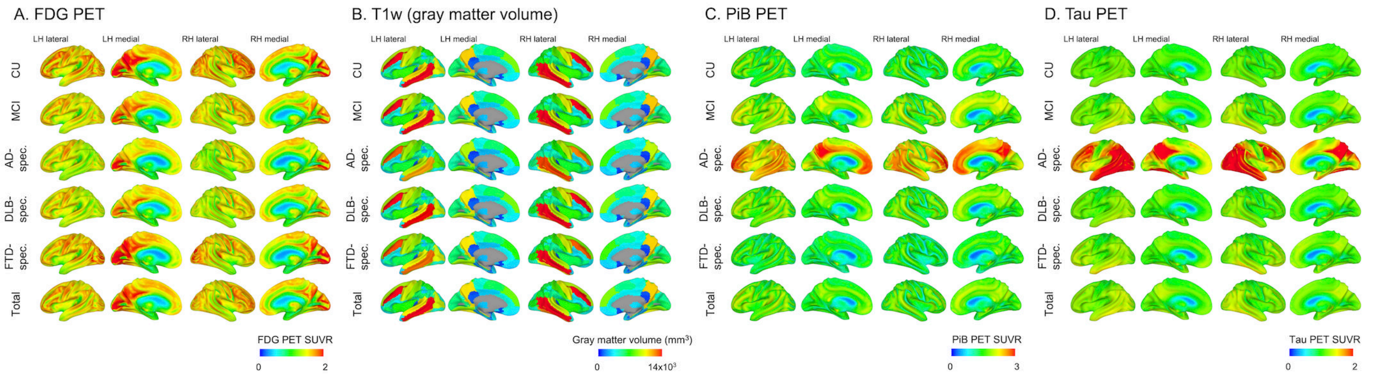

Supplementary figure 2. Diagnostic group-averaged images. (A) FDG PET, (B) T1w, (C) PiB PET and (D) Tau PET.

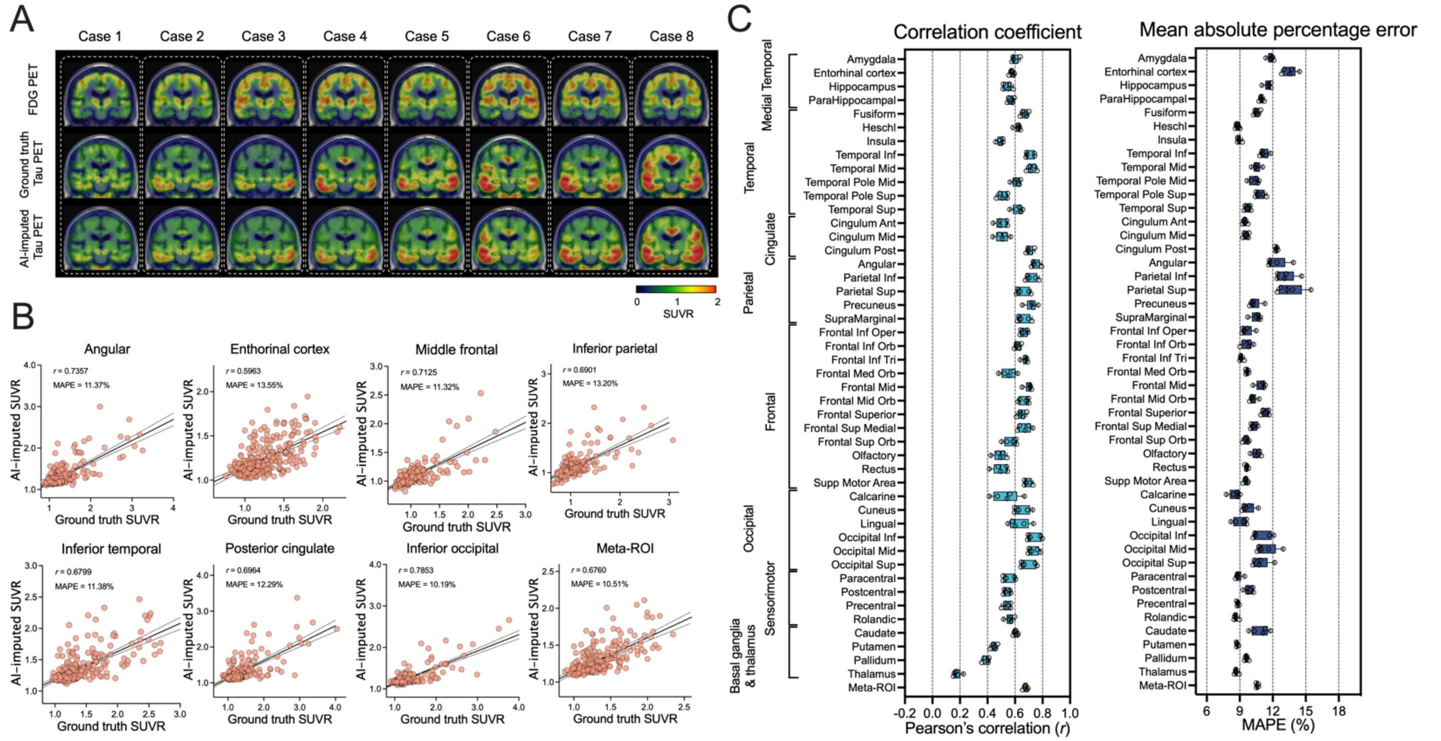

Supplementary figure 3. Testing the FDG-based model on the ADNI dataset. (A) Eight representative cases with original FDG-PET, ground truth tau PET and AI-imputed tau PET. (B) Scatter plots of ground truth tau PET and AI-imputed tau PET from seven representative ROIs and meta-ROI. *r* indicates the Pearson's correlation coefficient and MAPE indicates mean absolute percentage error. Linear regression (black line) and 95% confidence bands (dotted lines) are shown. (C) The correlation coefficient and MAPE from 46 ROIs and meta-ROI is summarized in a box plot. The yellow-colored box depicts the meta-ROI result.

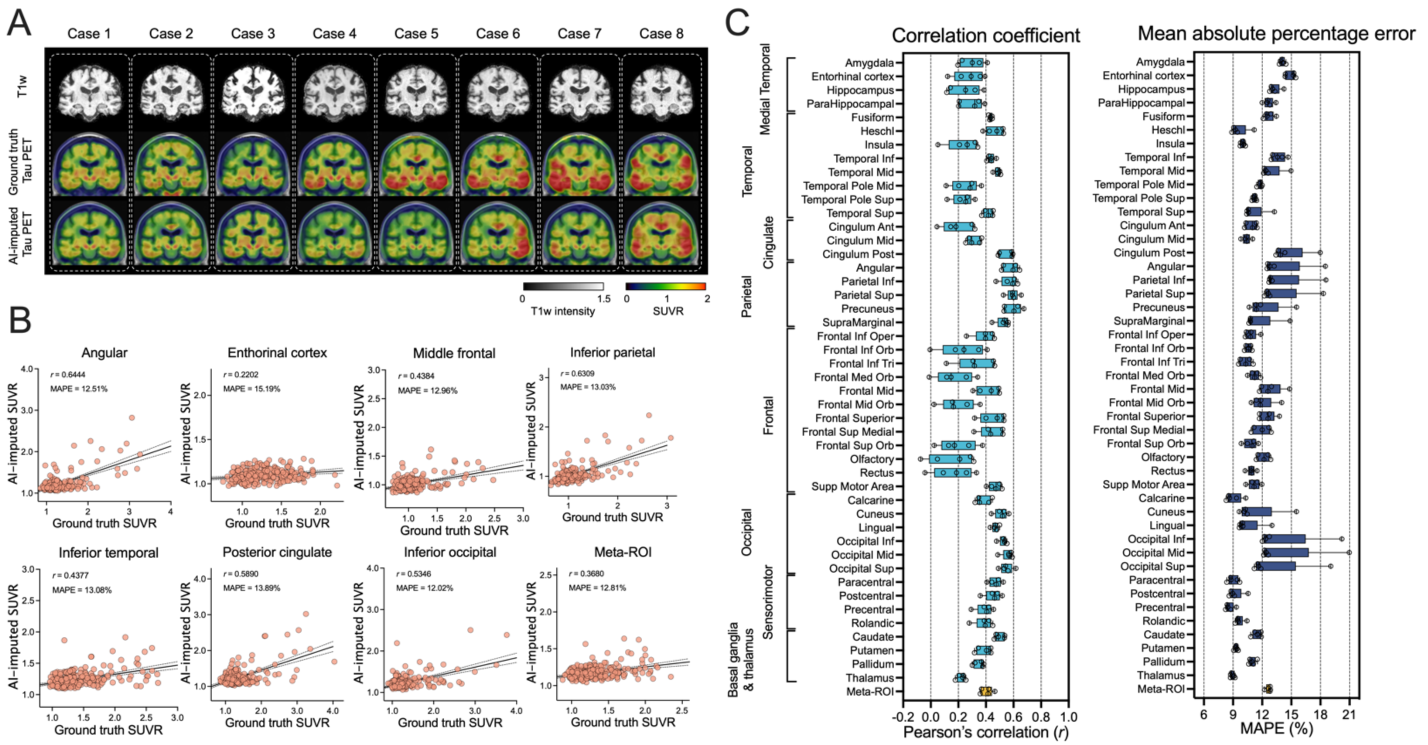

Supplementary figure 4. Testing the T1w-based model on the ADNI dataset. (A) Eight representative cases with original T1w, ground truth tau PET and AI-imputed tau PET. (B) Scatter plots of ground truth tau PET and AI-imputed tau PET from seven representative ROIs and meta-ROI. *r* indicates the Pearson's correlation coefficient and MAPE indicates mean absolute percentage error. Linear regression (black line) and 95% confidence bands (dotted lines) are shown. (C) The correlation coefficient and MAPE from 46 ROIs and meta-ROI is summarized in a box plot. The yellow-colored box depicts the meta-ROI result.

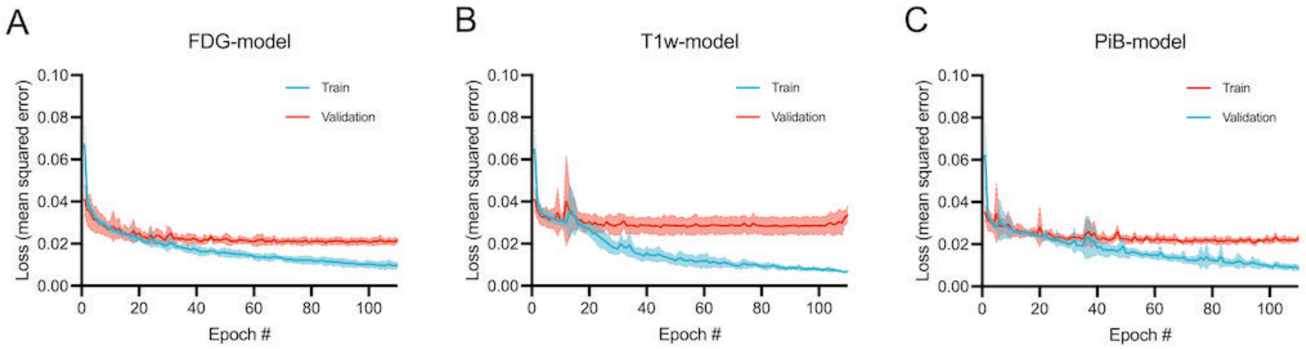

Supplementary figure 5. Train and validation loss as the epoch increases (A) FDG-model, (B) T1w-model, (C) PiB-model. Solid line and shaded area show an average and standard deviation of five folds, respectively.

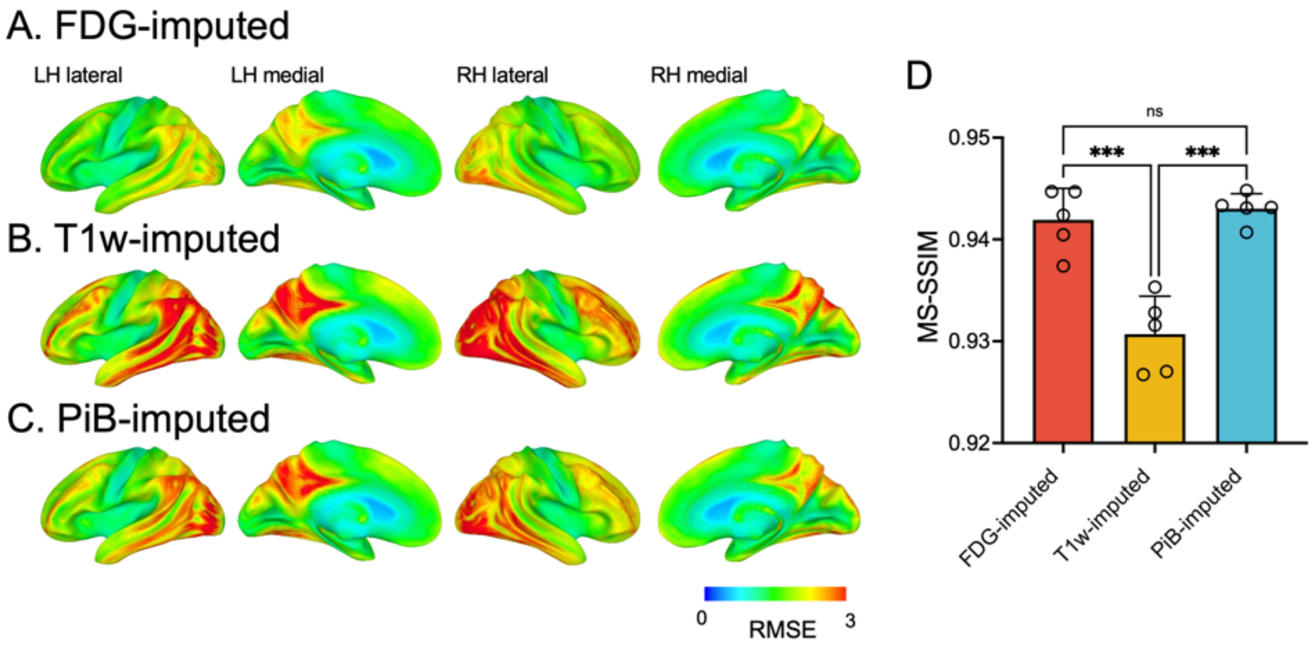

Supplementary figure 6. Voxel-wise evaluation of the model's performance. (A-C) 3D rendered images of the voxel-wise root mean squared error (RMSE) map. (D) multi-scale structural similarity index for FDG-, T1w-, and PiB-based models. Statistical significance was evaluated with one-way ANOVA and Holm-Sidak post-hoc test, *** $p<0.001$.

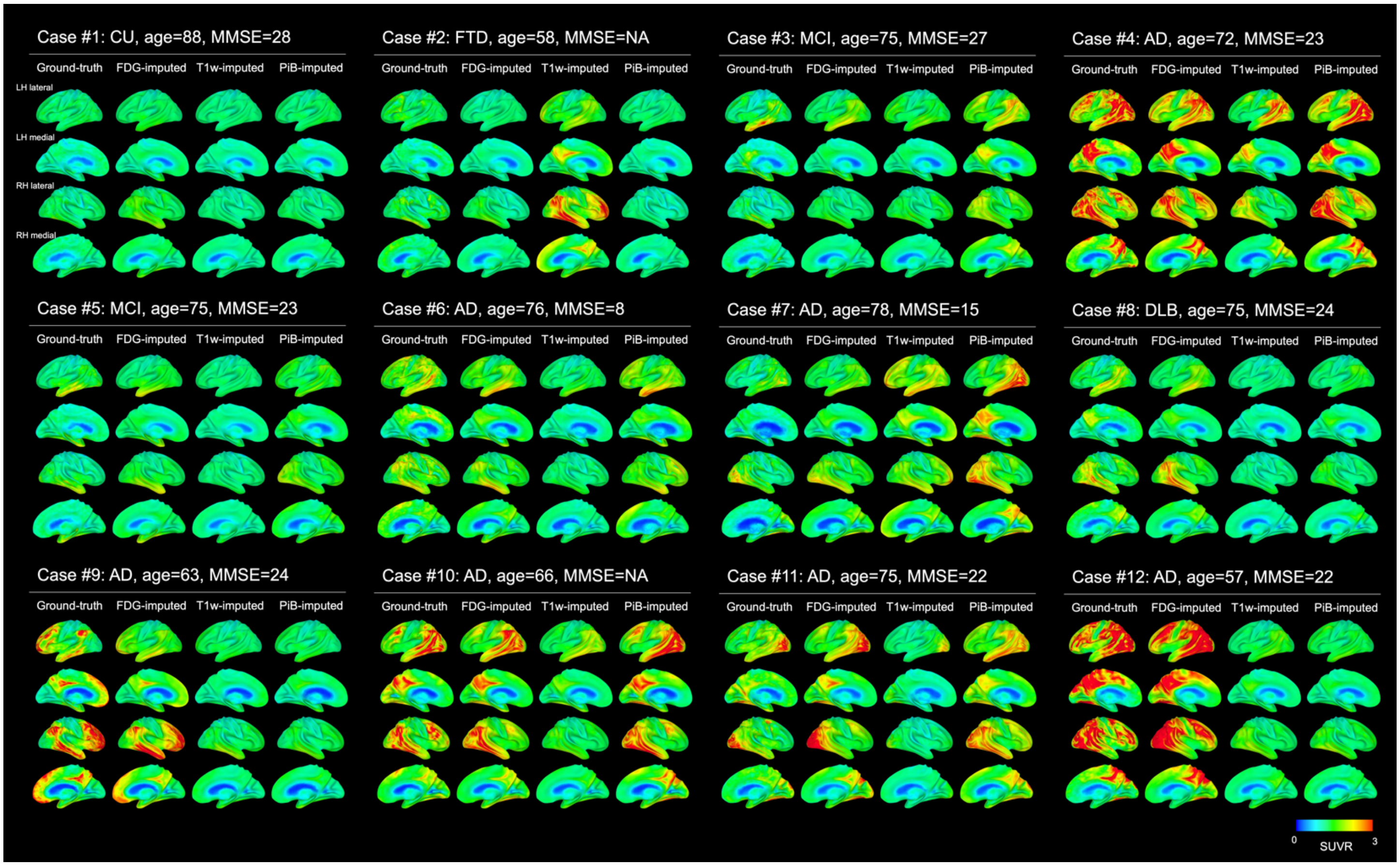

Supplementary figure 7. Comparisons of performance among the AI-imputed tau PET and ground truth tau PET in 3-dimensional stereotactic surface projection images.

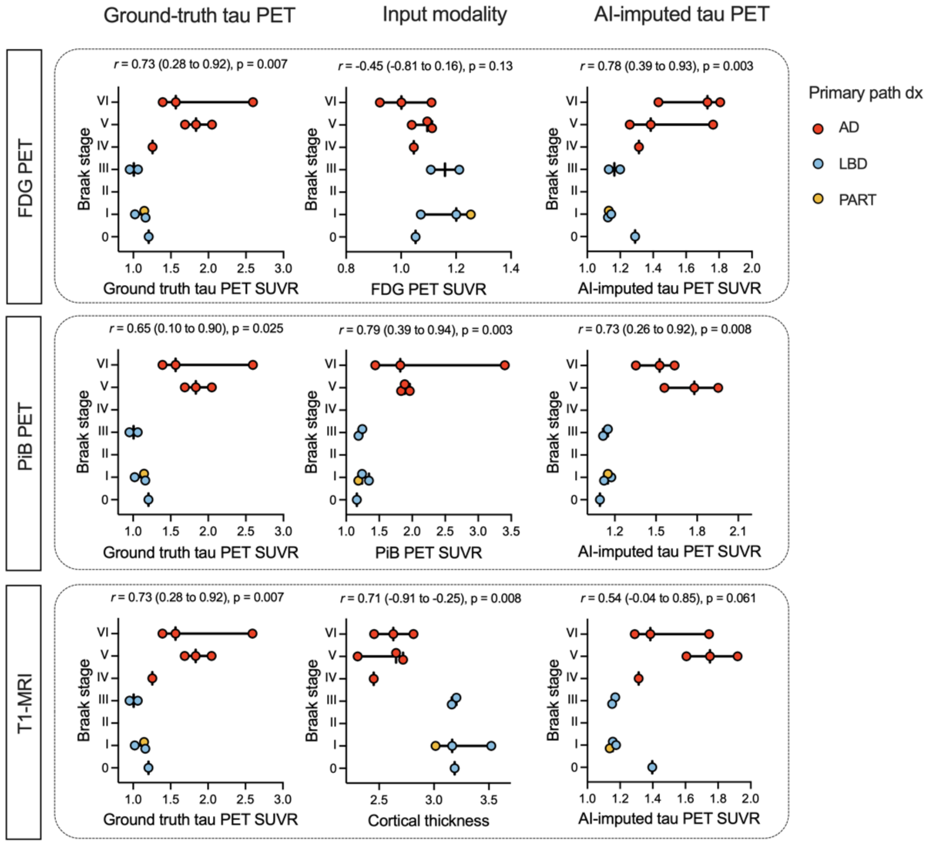

Supplementary figure 8. AI-imputed tau PET and Braak stage. Each participant's primary pathological diagnosis is visualized as different colors of dots. r, Spearman's correlation coefficient; p, correlation test p-value. Abbreviations: AD, Alzheimer's disease; LBD, Lewy body disorders; PART, primary age-related tauopathy.

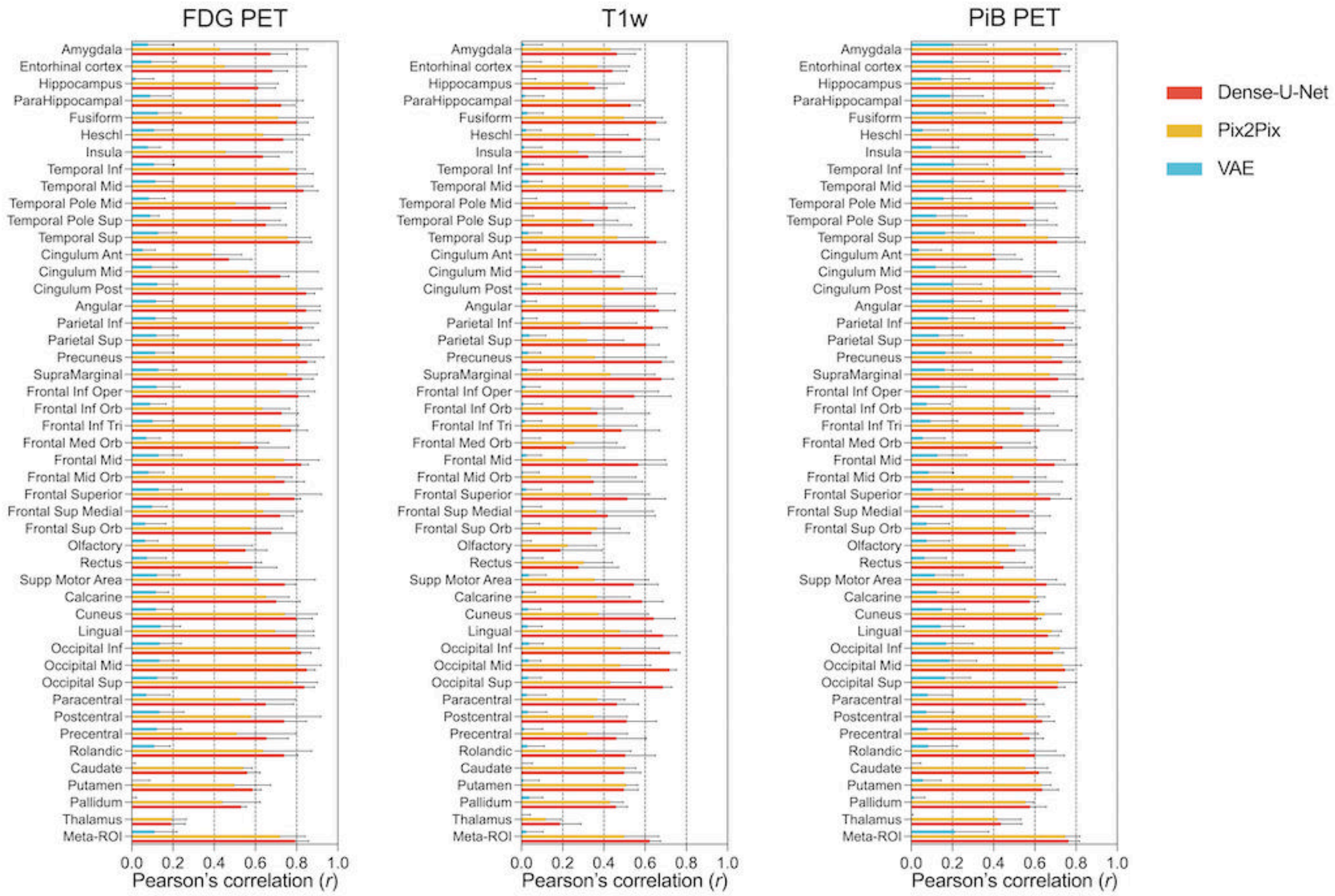

Supplementary figure 9. Regional correlation coefficient from the different architectures.

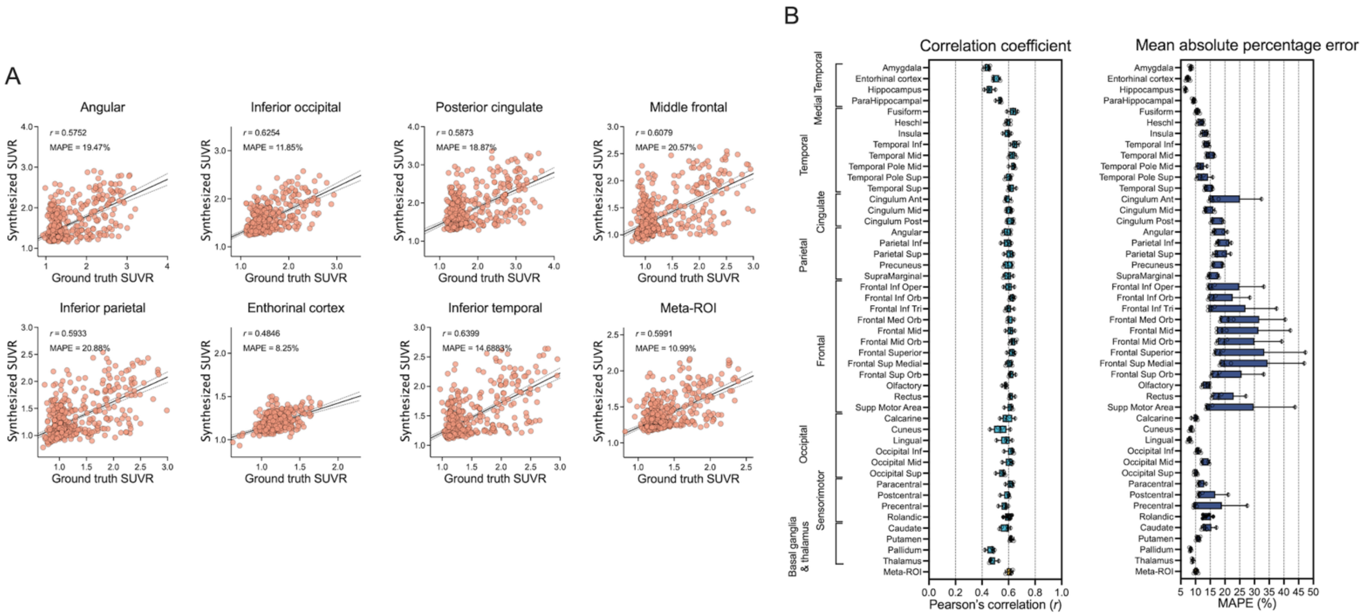

Supplementary figure 10. FDG PET based PiB PET synthesis results. (A) Scatter plots between ground-truth PiB-PET and AI-imputed PiB-PET from seven representative ROIs and meta-ROI. r indicates the Pearson's correlation coefficient and MAPE indicates mean absolute percentage error. Linear regression (black line) and 95% confidence bands (dotted lines) are shown. (B) The mean correlation coefficient and MAPE of five folds from 46 ROIs and meta-ROI is summarized in the box plots. The yellow-colored box depicts the meta-ROI result. Open circles indicate different folds.

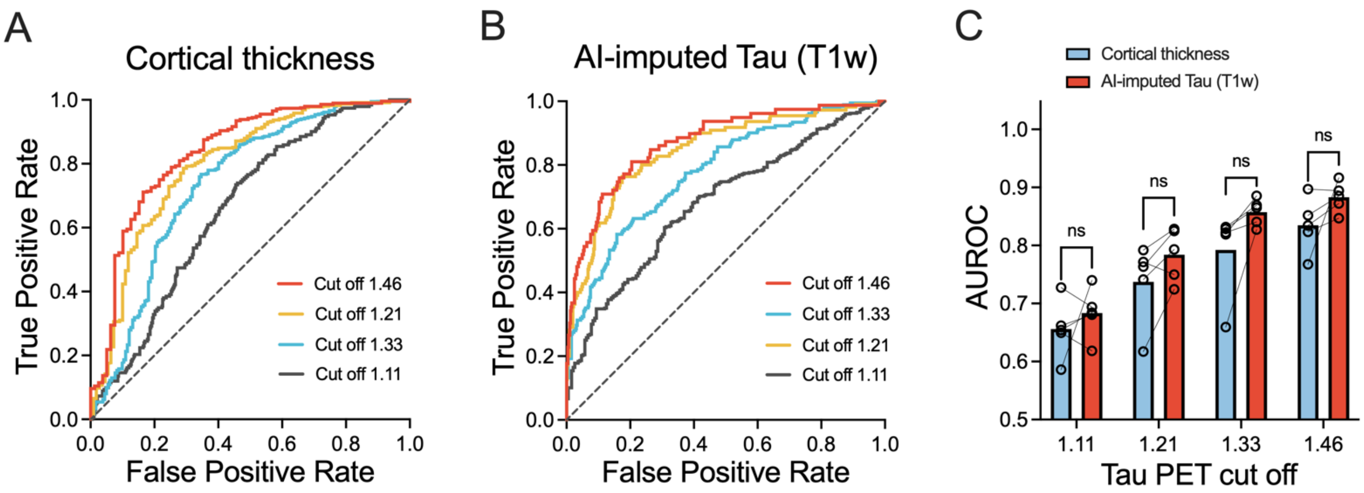

Supplementary figure 11. ROC analysis for the Siemens cohort. For cortical thickness, the participants who had Siemens MRI scans were separately analyzed. (A) Cortical thickness (B) T1W-based AI-imputed tau PET (C) AUROC comparison between the cortical thickness and T1W-based AI-imputed tau PET. Statistical significance was assessed with two-way ANOVA and Holm-Sidak post hoc comparison.

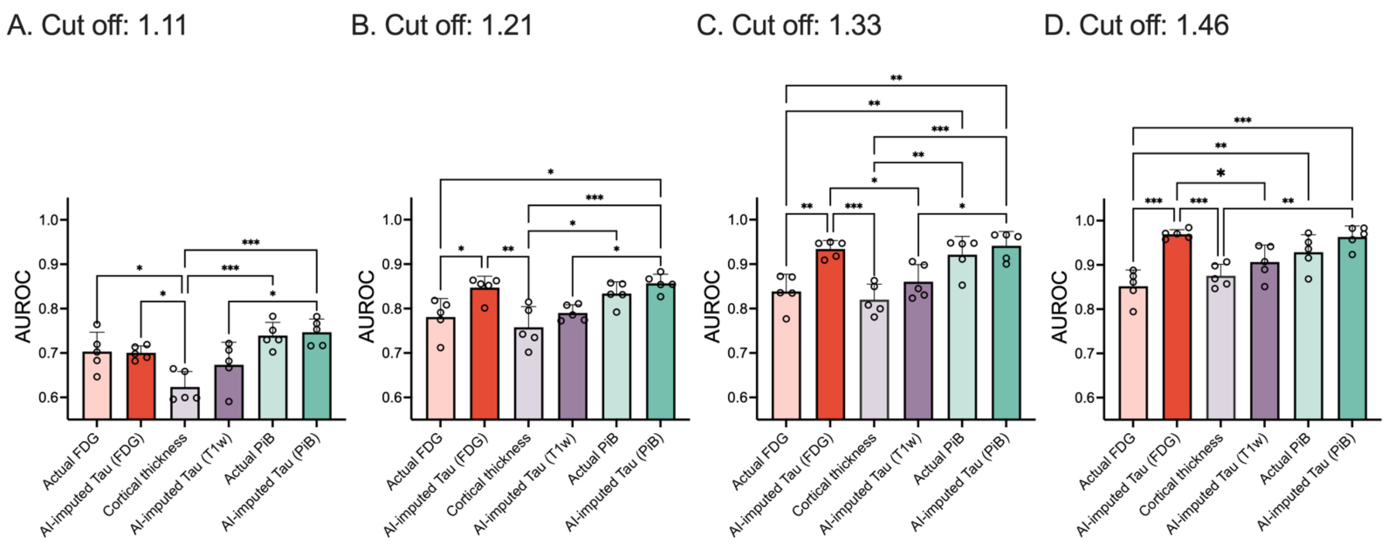

Supplementary figure 12. AUROC comparisons for tau positivity. The prediction accuracies (AUROC) for tau positivity were compared among six predictors for (A) 1.11, (B) 1.21, (C) 1.33, and (D) 1.46 cutoff values. Statistical significance was assessed with one-way ANOVA and Holm-Sidak post hoc comparison. Error bars indicate standard deviation. * $p<0.05$, ** $p<0.005$, *** $p<0.001$.

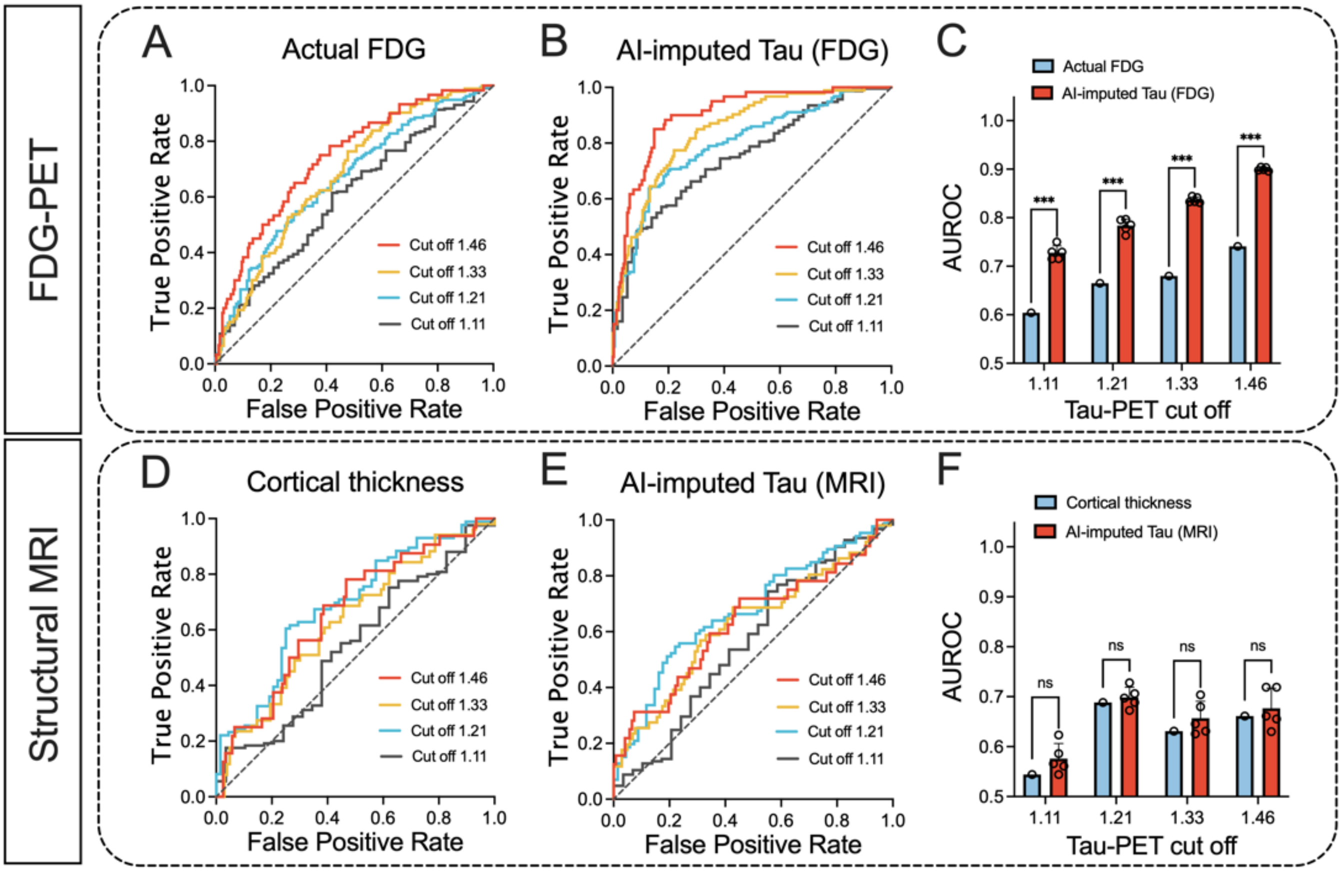

Supplementary figure 13. ROC curves showing the classification performance on the tau positivity tested on ADNI dataset. The tau positivity obtained from the ground-truth tau PET using four different meta-ROI cutoff thresholds (1.11, 1.21, 1.33, and 1.46) were predicted. (A) Actual FDG-PET (B) FDG-based AI-imputed tau PET (C) AUROC comparison between the original FDG and FDG-based AI-imputed tau PET. (D) Cortical thickness from the cohort who had Siemens scans (E) T1W-based AI-imputed tau PET from the cohort who had Siemens scans (F) AUROC comparison between the cortical thickness and T1W-based AI-imputed tau PET. Statistical significance was assessed with two-way ANOVA and Holm-Sidak post hoc comparisons. *** $p<0.001$.

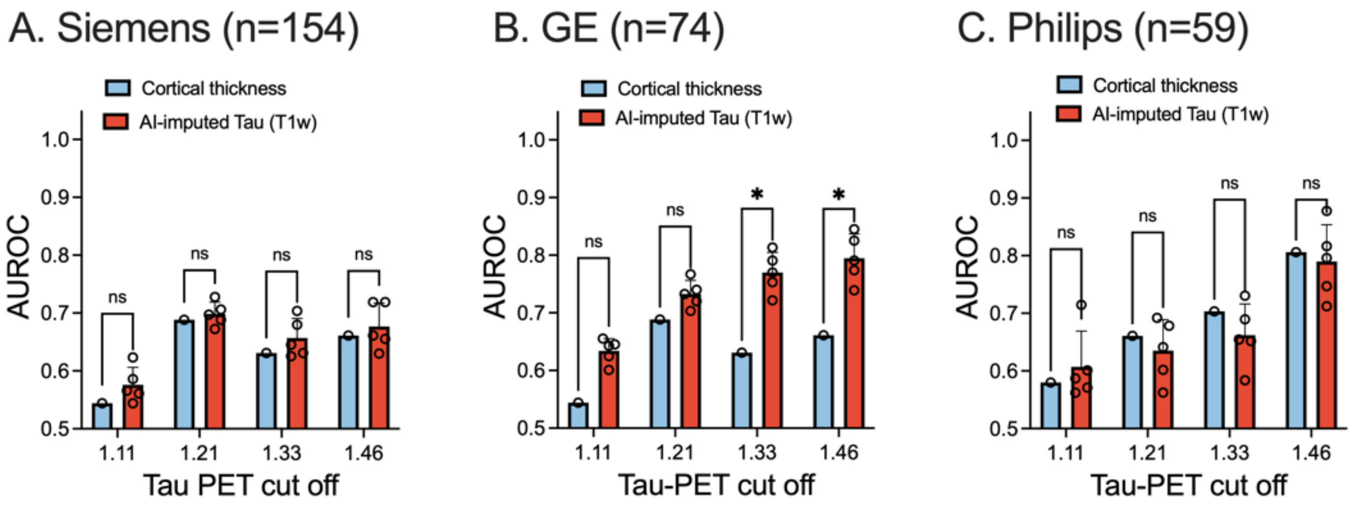

Supplementary figure 14. AUROC comparisons for different MRI manufacturers tested on ADNI dataset. The prediction accuracy (AUROC) using four different meta-ROI cutoff thresholds (1.11, 1.21, 1.33, and 1.46) for different MRI manufacturers: (A) The Siemens, (B) GE, and (C) Philips. Statistical significance was assessed with two-way ANOVA and Holm-Sidak post hoc comparisons. * $p<0.05$.

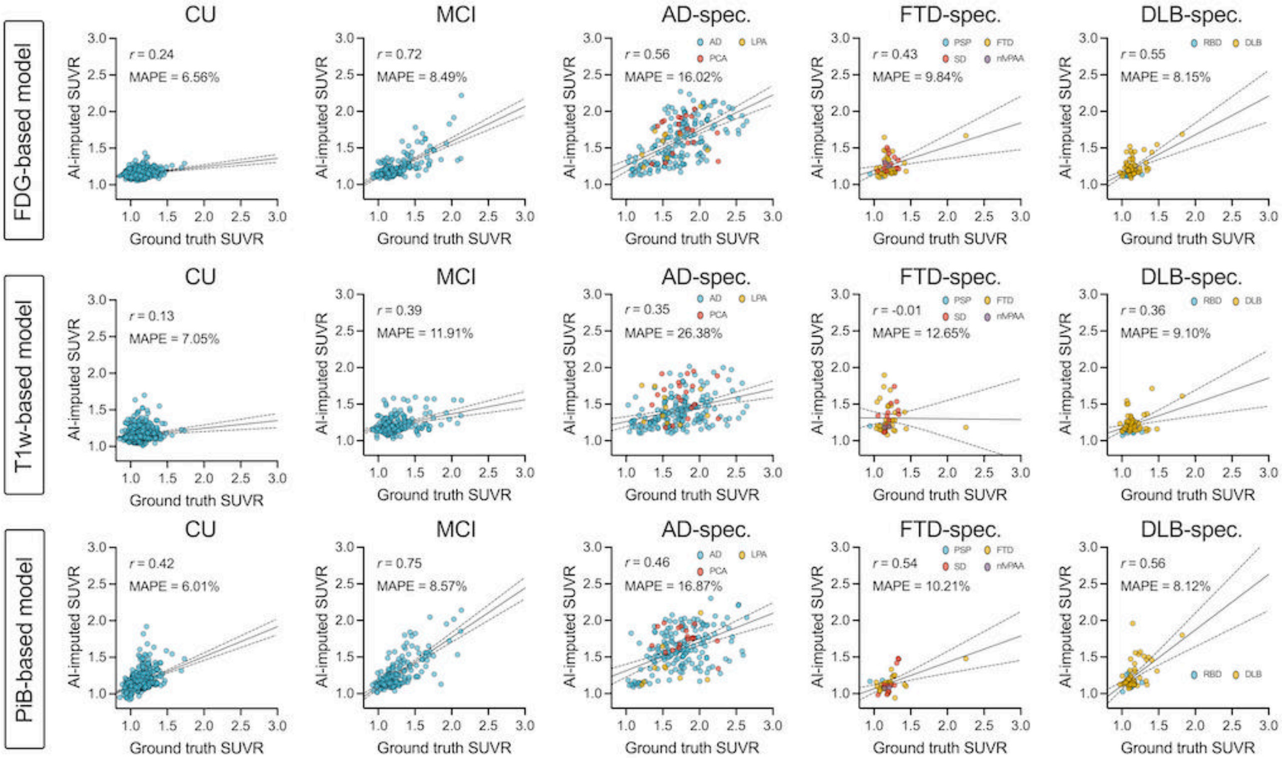

Supplementary figure 15. Disease group specific scatter plots between the ground truth SUVR vs. AI-imputed SUVR.

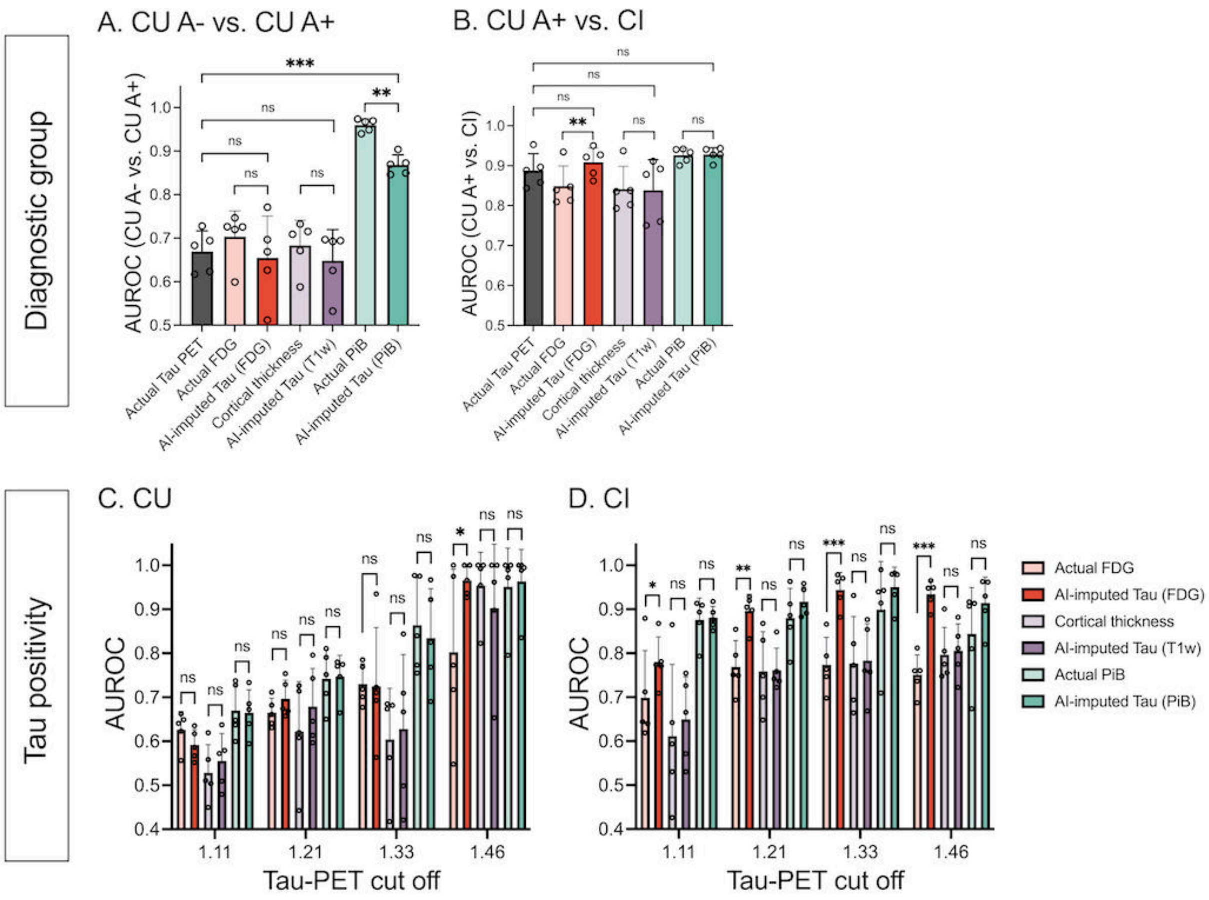

Supplementary figure 16. Evaluation of the model's performance at different disease stages. (A and B) ROC analysis for classifying the diagnostic groups: CU A- vs. CU A+ (A) and CU A+ vs. CI (B). (C and D) ROC analysis for predicting tau positivity for CU individuals (C) and CI individuals including those with MCI and AD (D). Statistical significance was assessed using a two-sample t-test. Open circles represent different folds.

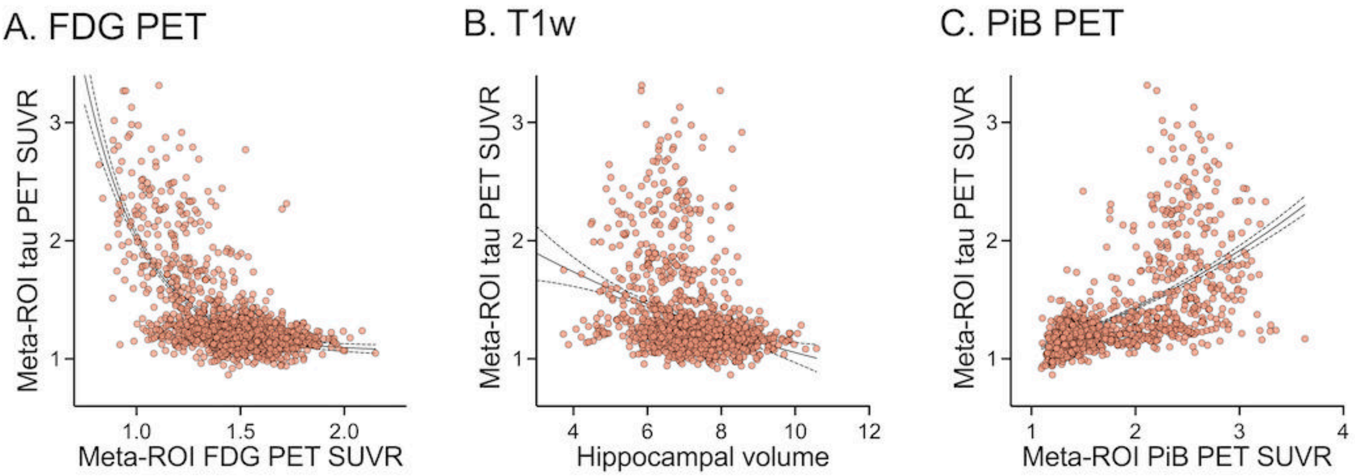

Supplementary figure 17. Associations between the input modality and tau PET meta-ROI SUVR. (A) FDG PET, (B) T1w, (C) PiB PET.

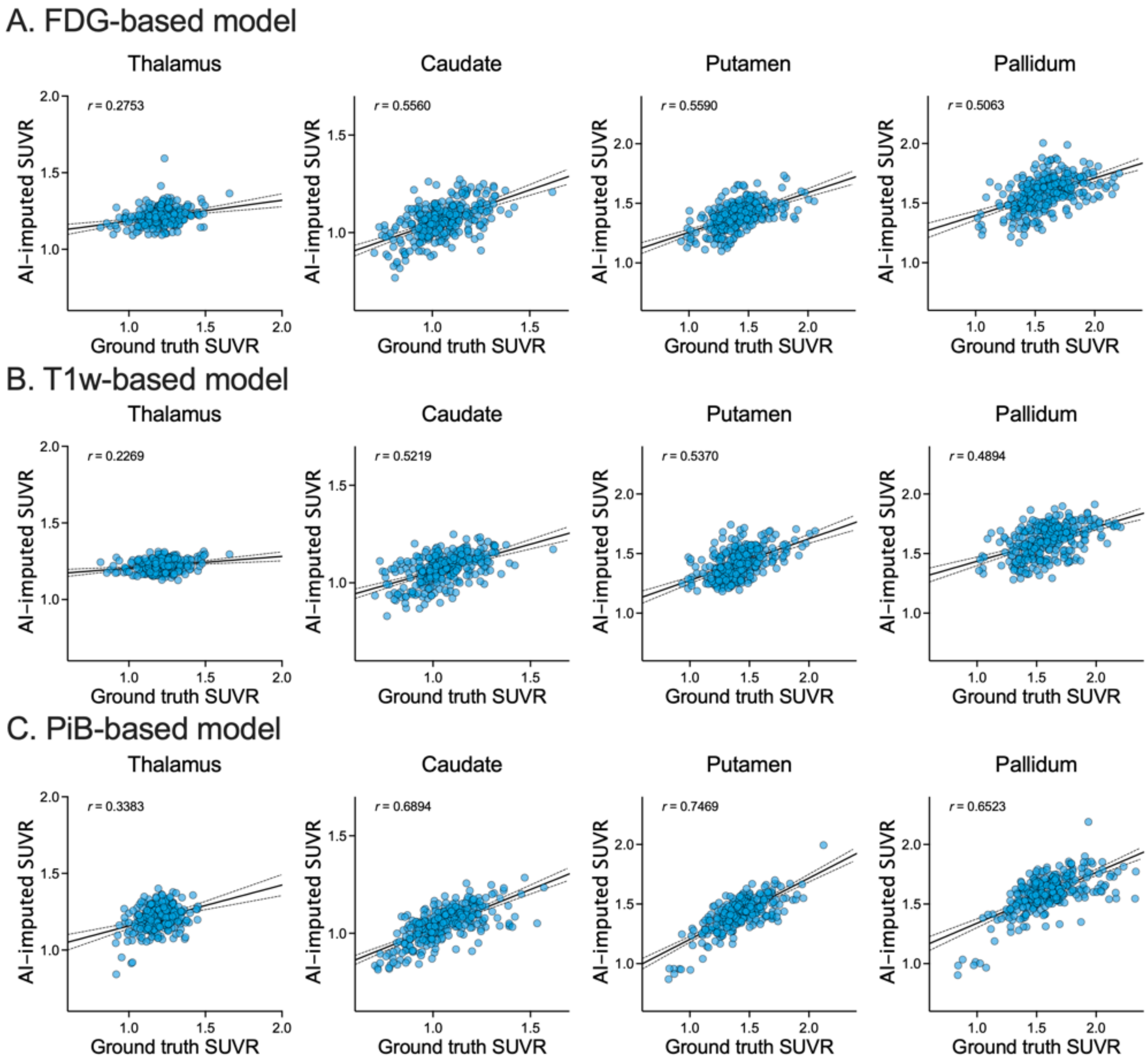

Supplementary figure 18. AI-imputed SUVR of regions of off-target bindings. r indicates Pearson's correlation.

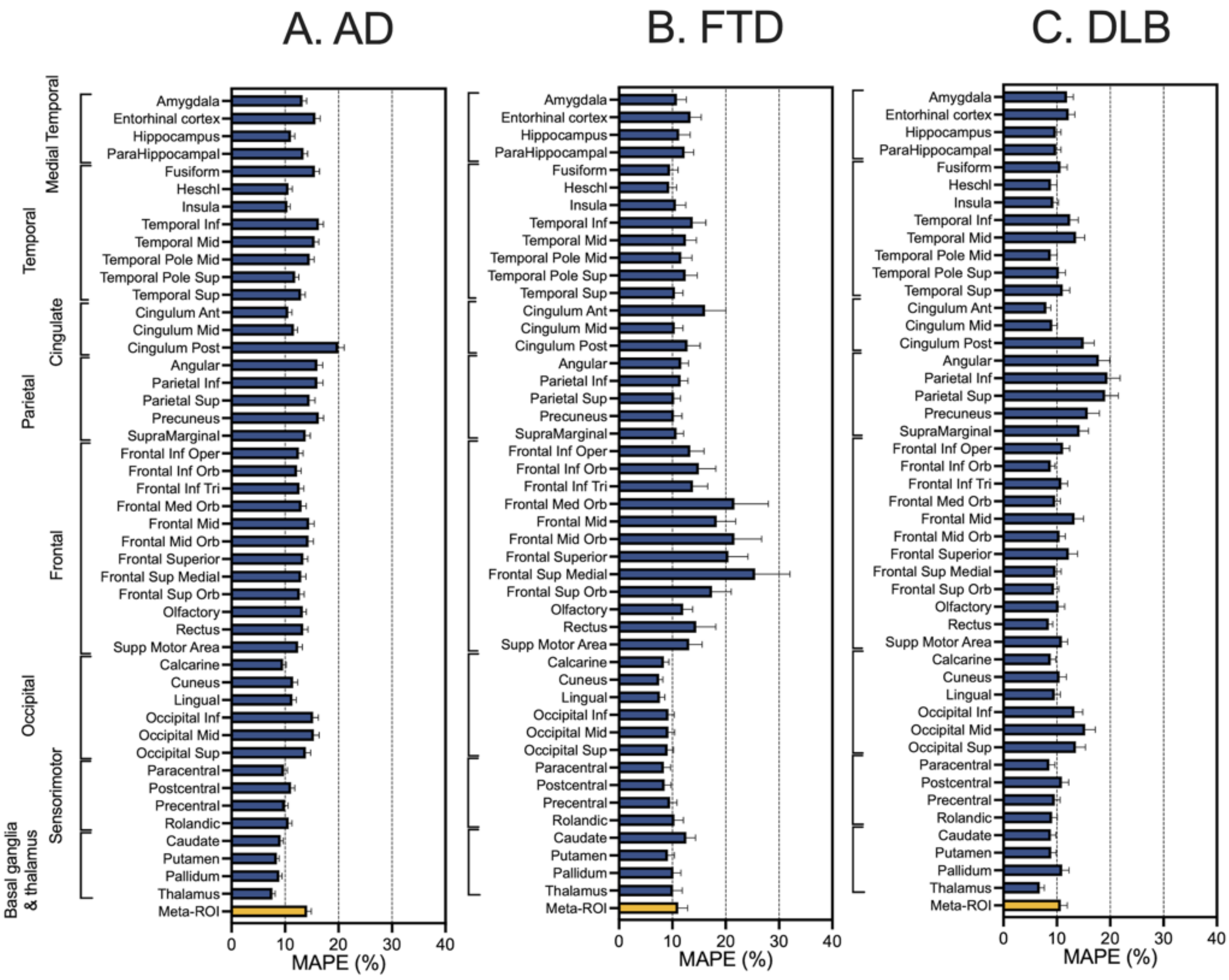

Supplementary figure 19. Regional MAPE distribution for FDG-based AI-imputed tau PET for AD, FTD, and DLB diagnostic groups.
