# Supplementary material for: Synthesizing images of tau pathology from cross-modal neuroimaging using deep learning
Source: Brain. 2023 Oct 7;147(3):980–95. doi: 10.1093/brain/awad346 (PMC10907092; doi:10.1093/brain/awad346)
Supplement: awad346_Supplementary_Data [file awad346_supplementary_data.zip › brain-2023-00368-File011.pdf]

Supplementary Table 1. ADNI Cohort Demographics

Characteristic	Clinical Diagnosis		
	Normal	MCI	Dementia
N (%)	15 (5.21)	205 (71.18)	68 (23.61)
Age, median (min max), years	68 (56 89)	75 (56 92)	77.5 (56 92)
Male sex, n (%)	7 (46.67)	117 (57.07)	39 (57.35)
Education, median (IQR), years	16 (14 18)	16 (14 18)	16 (13.5 18)
Clinical Dementia Rating Scale-Sum of Boxes, median	0 (0 0)	1 (0.5 2)	4.5 (3.5 5.5)
Meta-ROI FDG PET SUVR, median (IQR)	1.27 (1.24 1.29)	1.20 (1.13 1.27)	1.10 (1.03 1.17)
Meta-ROI Tau PET SUVR, median (IQR)	1.12 (1.06 1.15)	1.20 (1.12 1.33)	1.45 (1.26 1.67)

Supplementary Table 2. Demographics for FDG/MRI model.

	Fold 1				Fold 2				Fold 3				Fold 4				Fold 5			
	**Train (N=903)**	**Validation (N=294)**	**Test (N=308)**	**P value**	**Train (N=868)**	**Validation (N=343)**	**Test (N=294)**	**P value**	**Train (N=876)**	**Validation (N=286)**	**Test (N=343)**	**P value**	**Train (N=945)**	**Validation (N=274)**	**Test (N=286)**	**P value**	**Train (N=923)**	**Validation (N=308)**	**Test (N=274)**	**P value**
Age[a] Mean (SD), years	67 (13)	69 (11)	67 (12)	0.387	68 (12)	67 (13)	69 (11)	0.116	68 (12)	68 (12)	67 (13)	0.163	67 (12)	68 (13)	68 (12)	0.537	68 (12)	67 (12)	68 (13)	0.558
Male[b] N (%)	511 (57%)	160 (54%)	183 (59%)	0.461	483 (56%)	211 (62%)	160 (54%)	0.119	485 (55%)	158 (55%)	211 (62%)	0.127	554 (59%)	142 (52%)	158 (55%)	0.115	529 (57%)	183 (59%)	142 (52%)	0.156
APOE e4 carrier[b] N (%) {N-Miss}	303 (35%) {49}	110 (39%) {12}	97 (32%) {7}	0.232	282 (34%) {38}	118 (36%) {18}	110 (39%) {12}	0.078	297 (35%) {34}	95 (35%) {16}	118 (36%) {18}	0.94	325 (36%) {37}	90 (35%) {15}	95 (35%) {16}	0.947	323 (37%) {46}	97 (32%) {7}	90 (35%) {15}	0.341
Diagnosis[b] N (%)																				
CU	535 (59%)	169 (57%)	186 (60%)		521 (60%)	200 (58%)	169 (57%)		513 (59%)	177 (62%)	200 (58%)		555 (59%)	158 (58%)	177 (62%)		546 (59%)	186 (60%)	158 (58%)	
MCI	132 (15%)	36 (12%)	40 (13%)		114 (13%)	58 (17%)	36 (12%)		113 (13%)	37 (13%)	58 (17%)		134 (14%)	37 (14%)	37 (13%)		131 (14%)	40 (13%)	37 (14%)	
AD-spec.	109 (12%)	52 (18%)	33 (11%)	**0.007**	97 (11%)	45 (13%)	52 (18%)	**0.004**	122 (14%)	27 (9%)	45 (13%)	0.209	130 (14%)	37 (14%)	27 (9%)	0.207	124 (13%)	33 (11%)	37 (14%)	0.441
DLB-spec.	52 (6%)	9 (3%)	13 (4%)		51 (6%)	14 (4%)	9 (3%)		40 (5%)	20 (7%)	14 (4%)		36 (4%)	18 (7%)	20 (7%)		43 (5%)	13 (4%)	18 (7%)	
FTD-spec.	32 (4%)	3 (1%)	14 (5%)		34 (4%)	12 (3%)	3 (1%)		29 (3%)	8 (3%)	12 (3%)		29 (3%)	12 (4%)	8 (3%)		23 (2%)	14 (5%)	12 (4%)	
Other	43 (5%)	25 (9%)	22 (7%)		51 (6%)	14 (4%)	25 (9%)		59 (7%)	17 (6%)	14 (4%)		61 (6%)	12 (4%)	17 (6%)		56 (6%)	22 (7%)	12 (4%)	
MMSE[a], Mean (SD) {N-Miss}	27 (4) {44}	27 (4) {20}	27 (3) {20}	0.419	27 (4) {48}	27 (4) {16}	27 (4) {20}	0.765	27 (4) {52}	27 (3) {16}	27 (4) {16}	0.542	27 (4) {56}	27 (4) {12}	27 (3) {16}	0.213	27 (4) {52}	27 (3) {20}	27 (4) {12}	0.185
Aβ positivity, % {N-Miss}	42.15% {22}	49.83% {7}	40% {8}	**0.037**	41.04% {20}	43.84% {10}	49.83% {7}	**0.034**	44.16% {20}	40.5% {7}	43.84% {10}	0.554	44.46% {25}	42.75% {5}	40.5% {7}	0.492	44.72% {24}	40% {8}	42.75% {5}	0.351
Tau PET meta-ROI SUVR[c,] Median (Q1 Q3)	1.21 (1.15, 1.31)	1.22 (1.15, 1.34)	1.2 (1.15, 1.29)	0.065	1.21 (1.15, 1.30)	1.22 (1.14, 1.34)	1.22 (1.15, 1.34)	0.259	1.21 (1.15, 1.31)	1.2 (1.13, 1.28)	1.22 (1.14, 1.34)	0.303	1.21 (1.15, 1.32)	1.22 (1.17, 1.31)	1.2 (1.13, 1.28)	0.124	1.21 (1.14, 1.32)	1.2 (1.15, 1.29)	1.22 (1.17, 1.31)	**0.034**

Statistical test was performed within each fold.

[a]Linear Model ANOVA,

[b]Pearson's Chi-squared test,

[c]Median test.

Abbreviations: CU: Clinically Unimpaired; MCI: Mild Cognitive Impairment; AD: Alzheimer's Dementia; DLB: Dementia with Lewy Bodies; FTD: Frontotemporal Dementia; MMSE: Mini-Mental State Examinations.

Supplementary Table 3. Demographics for PiB model.

	**Fold 1**				**Fold 2**				**Fold 3**				**Fold 4**				**Fold 5**			
	**Train (N=885)**	**Validation (N=301)**	**Test (N=293)**	**P value**	**Train (N=859)**	**Validation (N=319)**	**Test (N=301)**	**P value**	**Train (N=876)**	**Validation (N=284)**	**Test (N=319)**	**P value**	**Train (N=913)**	**Validation (N=282)**	**Test (N=284)**	**P value**	**Train (N=904)**	**Validation (N=293)**	**Test (N=282)**	**P value**
Age[a] Mean (SD)	67 (13)	69 (11)	67 (12)	0.387	67 (12)	69 (12)	68 (12)	0.21	68 (12)	66 (12)	69 (12)	**0.02**	68 (12)	67 (13)	66 (12)	**0.012**	68 (12)	69 (12)	67 (13)	0.378
Male[b] N (%)	496 (56%)	171 (57%)	173 (59%)	0.668	486 (57%)	183 (57%)	171 (57%)	0.971	493 (56%)	164 (58%)	183 (57%)	0.886	527 (58%)	149 (53%)	164 (58%)	0.329	518 (57%)	173 (59%)	149 (53%)	0.287
APOE e4 carrier[b] N (%) {N-Miss}	303 (36%) {46}	105 (36%) {9}	96 (34%) {13}	0.854	288 (35%) {44}	111 (37%) {15}	105 (36%) {9}	0.931	293 (35%) {35}	100 (38%) {18}	111 (37%) {15}	0.679	312 (36%) {37}	92 (34%) {13}	100 (38%) {18}	0.711	316 (37%) {42}	96 (34%) {13}	92 (34%) {13}	0.653
Diagnosis[b] N (%)																				
CU	538 (61%)	166 (55%)	183 (62%)		544 (63%)	177 (55%)	166 (55%)		531 (61%)	179 (63%)	177 (55%)		526 (58%)	182 (65%)	179 (63%)		522 (58%)	183 (62%)	182 (65%)	
MCI	112 (13%)	42 (14%)	50 (17%)	**0.008**	112 (13%)	50 (16%)	42 (14%)	**0.018**	124 (14%)	30 (11%)	50 (16%)	0.333	142 (16%)	32 (11%)	30 (11%)	**0.026**	122 (13%)	50 (17%)	32 (11%)	**0.017**
AD-spec.	125 (14%)	38 (13%)	27 (9%)		111 (13%)	41 (13%)	38 (13%)		107 (12%)	42 (15%)	41 (13%)		106 (12%)	42 (15%)	42 (15%)		121 (13%)	27 (9%)	42 (15%)	
DLB-spec.	40 (5%)	25 (8%)	7 (2%)		29 (3%)	18 (6%)	25 (8%)		41 (5%)	13 (5%)	18 (6%)		50 (5%)	9 (3%)	13 (5%)		56 (6%)	7 (2%)	9 (3%)	
FTD-spec.	18 (2%)	12 (4%)	10 (3%)		18 (2%)	10 (3%)	12 (4%)		27 (3%)	3 (1%)	10 (3%)		32 (4%)	5 (2%)	3 (1%)		25 (3%)	10 (3%)	5 (2%)	
Other	52 (6%)	18 (6%)	16 (5%)		45 (5%)	23 (7%)	18 (6%)		46 (5%)	17 (6%)	23 (7%)		57 (6%)	12 (4%)	17 (6%)		58 (6%)	16 (5%)	12 (4%)	
MMSE[a], Mean (SD) {N-Miss}	27 (4) {37}	27 (4) {23}	27 (3) {15}	0.058	27 (3) {41}	27 (4) {11}	27 (4) {23}	0.024	27 (4) {44}	27 (3) {20}	27 (4) {11}	0.152	27 (4) {49}	27 (4) {6}	27 (3) {20}	0.17	27 (4) {54}	27 (3) {15}	27 (4) {6}	0.3
Aβ positivity, %	42.17%	47.18%	43.15%	0.318	42.09%	43.31%	47.18%	0.309	44.20%	40.99%	43.31%	0.638	44.54%	42.09%	40.99%	0.51	43.88%	43.15%	42.09%	0.867
Tau PET meta-ROI SUVR[c,] Median (Q1 Q3)	1.21 (1.15, 1.31)	1.21 (1.15, 1.33)	1.21 (1.15, 1.30)	0.77	1.21 (1.14, 1.30)	1.22 (1.15, 1.34)	1.21 (1.15, 1.33)	0.806	1.21 (1.15, 1.31)	1.21 (1.14, 1.30)	1.22 (1.15, 1.34)	0.83	1.21 (1.15, 1.32)	1.21 (1.14, 1.30)	1.21 (1.14, 1.30)	0.912	1.21 (1.15, 1.32)	1.21 (1.15, 1.30)	1.21 (1.14, 1.30)	0.663

Statistical test was performed within each fold.

[a]Linear Model ANOVA,

[b]Pearson's Chi-squared test,

[c]Median test.

Abbreviations: CU: Clinically Unimpaired; MCI: Mild Cognitive Impairment; AD: Alzheimer's Dementia; DLB: Dementia with Lewy Bodies; FTD: Frontotemporal Dementia; MMSE: Mini-Mental State Examinations.

Supplementary Table 4. Comparisons of model's performance for different input modalities.

Comparison (A vs. B)	Mean rank difference (A-B	Summary	Adjusted P Value
FDG vs. T1w	120.91	***	<0.001
FDG vs. PiB	63.532	**	0.002
FDG vs. FDG+PiB	2.8723	ns	>0.99
FDG vs. FDG+T1w	11.851	ns	>0.99
FDG vs. PiB+T1w	61.191	**	0.004
T1w vs. PiB	-57.383	**	0.01
T1w vs. FDG+PiB	-118.04	***	<0.001
T1w vs. FDG+T1w	-109.06	***	<0.001
T1w vs. PiB+T1w	-59.723	**	0.006
PiB vs. FDG+PiB	-60.66	**	0.005
PiB vs. FDG+T1w	-51.681	*	0.03
PiB vs. PiB+T1w	-2.3404	ns	>0.99
FDG+PiB vs. FDG+T1w	8.9787	ns	>0.99
FDG+PiB vs. PiB+T1w	58.319	**	0.008
FDG+T1w vs. PiB+T1w	49.34	ns	0.05

The significance was tested by non-parametric Dunn's multiple comparisons test after one-way ANOVA.

Supplemetnary Table 5. Comparisons of model's performance for different architecture.

Input modality	Comparison (A vs. B)	Mean rank difference (A-B	Summary	Adjusted P Value
	Dense-U-Net vs. Pix2Pix	52.894	ns	0.32
FDG PET	Dense-U-Net vs. VAE	273.55	***	<0.001
	Pix2Pix vs. VAE	220.66	***	<0.001
	Dense-U-Net vs. Pix2Pix	63.489	ns	0.11
T1w	Dense-U-Net vs. VAE	215.06	***	<0.001
	Pix2Pix vs. VAE	151.57	***	<0.001
	Dense-U-Net vs. Pix2Pix	20.106	ns	>0.99
PiB PET	Dense-U-Net vs. VAE	211.51	***	<0.001
	Pix2Pix vs. VAE	191.4	***	<0.001

The significance was tested by non-parametric Dunn's multiple comparisons test after one-way ANOVA.

Supplementary Table 6. Comparisons of model's performance for different input modalities within each architecture.

Architecture	Comparison (A vs. B)	Mean rank difference (A-B	Summary	Adjusted P Value
Dense-U-Net	FDG PET vs. T1w	58.404	***	<0.001
	FDG PET vs. PiB PET	27.574	**	0.003
	T1w vs. PiB PET	-30.83	***	<0.001
Pix2Pix	FDG PET vs. T1w	64.426	***	<0.001
	FDG PET vs. PiB PET	4.7021	ns	>0.99
	T1w vs. PiB PET	-59.723	***	<0.001
VAE	FDG PET vs. T1w	52.362	***	<0.001
	FDG PET vs. PiB PET	-14.511	ns	0.26
	T1w vs. PiB PET	-66.872	***	<0.001

The significance was tested by non-parametric Dunn's multiple comparisons test after one-way ANOVA.
